# scRNA seq of an F1 cross of Marek’s disease resistant and susceptible chickens identifies allele specific expression signatures enriched in transcription modulators

**DOI:** 10.1038/s41598-025-86174-w

**Published:** 2025-01-29

**Authors:** Deborah Velez-Irizarry, Hans Cheng, Cari Hearn

**Affiliations:** USDA, Agricultural Research Service, US National Poultry Research Center, 934 College Station Road, Athens, GA 30605 USA

**Keywords:** Marek’s Disease Virus, Single cell RNA sequencing, Allele specific expression, Genetic resistance, Avian immune response, Immunogenetics, Viral infection

## Abstract

Marek’s disease (MD), a T cell lymphoma disease in chickens, is caused by the Marek’s disease virus (MDV) found ubiquitously in the poultry industry. Genetically resistant Line 6_3_ (L6) and susceptible Line 7_2_ (L7) chickens have been instrumental to research on avian immune system response to MDV infection. In this study we characterized molecular signatures unique to splenic immune cell types across different genetic backgrounds 6 days after infection. Using three populations, L6, L7, and an F1 cross between L6xL7, we evaluated the immune cell transcriptome of responding cell types using single cell RNA sequencing. Several MDV genes were found expressed mainly in cytotoxic T cells while ICP4 and MEQ MDV genes were expressed across infected cell types. Using the F1 we quantified allele specific expression (ASE) of biallelic SNPs and found biased expression of parental alleles specific to immune cell subtypes. We identified 22 SNPs with ASE in response to MDV infection mapped to gene rich regions surrounding 59 genes of critical importance for chromatin remodeling and transcriptional regulation. Histone deacetylase genes (HDAC1 and HDAC8) had increased expression of L6 alleles, while small nuclear RNA genes (SNORA68 and SNORA72) expressed higher levels of L7 alleles with infection in T cell subsets. SNPs with ASE also mapped genes important for an adequate immune response including GNLY (cytotoxic activity) and PDIA3 (component of MHC class I peptide loading complex), and genes known to promote viral replication (MCM5 and EIF3M). These results show that functional variants associated with susceptibility to MD may have a bigger impact in subsets of immune cell types, and by characterizing the transcriptomes of these subtypes we can unravel molecular signatures specific to MD genomic resistance.

**Supplementary Information:**

The online version contains supplementary material available at 10.1038/s41598-025-86174-w.

## Introduction

Marek’s disease virus (MDV) is a highly contagious alphaherpesvirus with widespread incidence in the poultry industry. The double stranded DNA virus (~ 175 kb genome) infects chickens and turkeys causing a lymphoproliferative disease known as Marek’s disease (MD). Infected and susceptible birds develop T cell lymphomas that cause peripheral nerve degeneration, partial or complete paralysis and immunosuppression^1–3^. Since the introduction of the first vaccine for MD in the 1960s, mass vaccination and development of newer more effective vaccines have been critical in the control of MD symptoms and in prevention of tumor formation. However, current and past vaccines do not provide sterilizing immunity to viral infection, therefore, MD-vaccinated birds can still shed viral particles. Consequently, MDV has remained a permissive pathogen in poultry farms. New disease outbreaks show more evolved virulent strains that evade vaccine protection with devastating losses to poultry farms^4–6^. Fundamental knowledge about the mechanisms involved in MDV immunity and a better understanding of the host genetic resistance have been pursued to develop more sustainable strategies to inform vaccine development and breeding programs.

Host genetic resistance is a complex trait and the use of experimental inbred lines that are resistant or susceptible to disease has contributed to identifying important resistance loci in the chicken for several important pathogens, including MDV^7–9^. The major histocompatibility complex (MHC) plays a critical role in antiviral immunity and genomic loci associated with enhanced genetic resistance to MDV have been successfully mapped to the MHC region^8^. While the MHC is important for antiviral and antitumor responses, it is unsurprisingly not the only locus contributing to MD resistance^10–17^, particularly as immune mechanisms contributing to disease resistance are regulated by complex interactions between a large number of genes and regulatory elements. The Avian Disease and Oncology Laboratory (ADOL) inbred chicken lines 6_3_ and 7_2_, which share the same B2 MHC haplotype, have been selected for MD resistance and susceptibility, respectively. These lines have proven to be a useful model for investigating non-MHC genetic mechanisms of resistance^8^. Several quantitative trait loci (QTL) have been successfully mapped for MD resistance using advanced crosses of highly inbred ADOL lines and partially inbred commercial lines that have been selected for resistance or susceptibility to MD. These studies have been instrumental to the discovery of candidate genes conferring MD resistance but the molecular mechanisms governing these signatures is still largely unknown. The mode of inheritance is greatly influenced by the genetic background of the population being investigated. Studies have shown that resistant alleles tend to be recessive in partially inbred commercial populations demonstrated by negative heterosis for resistance after six generations of intercrossing^12^. The contrary is seen in a first generation cross of highly inbred lines where resistance alleles tend to be dominantly inherited^15^. Moreover, QTL regions associated with resistance are not shared across commercial lines. These results reflect the complex nature of MD resistance that encompass pleiotropic interactions between multiple genes with potentially small effect size. Efforts to capture this pleiotropic effect have led to expression QTL analyses that identify single nucleotide polymorphisms (SNPs) that affect the expression of one or more genes. One form of eQTL analysis, known as allele specific expression analysis (ASE), measures the preferential expression of biallelic SNPs, a signature associated with epigenetic factors, such as cis-regulatory elements or methylation marks, regulating the expression of nearby genes. The mechanism underlying ASE depends on the location of the causative SNP and the type of regulatory element the SNP alters; for example a variant altering a cis-regulatory element in a non-coding region can cause differential binding of transcription factors while a coding variant can lead to a stop gain that targets the resulting mRNA for nonsense-mediated decay^18,19^. ASE screens have been widely adapted to the study of complex traits. Given the functional implications to phenotypic variation, ASE SNPs are amendable to marker assisted selection in agricultural species^19^. Intermatings of ADOL inbred lines that are homozygous for alleles conferring MD susceptibility have shown that allelic expression variation can be induced by the MDV viral oncogene Meq^20^ and SNPs exhibiting ASE in response to MDV infection can account for more than 80% of the genetic variance associated with MD genetic resistance^21^. Studies have shown that while transcriptional regulators of gene expression such as basal transcription factor genes map to ASE SNPs, genes regulating DNA replication and repair, and immune cell signaling pathways are also enriched for ASE SNPs^22^ in bulk sequencing of immune cell populations. Similar results were observed in CD4 T cell populations^23^. Therefore, genes associated with ASE SNPs should be key regulators of the transcriptional response to infection and underly important genetic differences in MD incidence.

Immunology research on host-pathogen interactions, particularly in agricultural species, presents multiple challenges due to an incomplete understanding of immune cell type frequencies and phenotypes, comparatively low number of cell markers characterized, and the limited availability of immunophenotyping reagents. Bulk RNA sequencing has greatly enhanced our understanding of the host’s immune response to viral infections, including MD^22,24–29^. However, bulk tissue profiling presents a homogenized picture of whole-organ responses, while identifying the transcriptional behavior of specific responding and infected cell types is likely to contribute additional knowledge of the cellular mechanisms involved in immune-protective or diseased states. Single-cell RNA sequencing (scRNA seq) offers a unique perspective on the response of distinct cell populations and enables the discovery of previously unknown cell types. It is therefore a valuable tool for the characterization of immune cells, especially in chickens where conventional tools are limited; scRNA seq has been used to identify chicken immune cell populations in peripheral blood mononuclear cells^30,31^, spleen^32^, bursa cells^33^, and inflammatory infiltrates as well as non-immune cell types in infectious bronchitis-infected kidney and trachea^34^. All these studies, except for one^31^, also examined cell-type-specific responses to viral infection in their datasets. Single cell sequencing enables the assessment of changes in molecular signaling and crosstalk, cell trafficking, population expansion and differentiation in response to infection.

Previously, our lab performed scRNAseq of splenic-derived cells from MD resistant Line 6_3_ (L6) and MD susceptible Line 7_2_ (L7) during MDV infection. Splenic tissues were chosen as they possess a unique microenvironment where humoral and cell-mediated responses interact to modulate immune cell activation during viral infection and contain the largest repository of mature lymphocytes compared to the other secondary lymphoid organs^35^. The study aimed to identify major immune cell subsets present in this peripheral lymphoid organ and characterize immunological differences in the response to infection at a cell-type level^32^. While this attempt was successful in providing the first single-cell immune profiling map of this organ in the chicken and identified novel infection response genes and pathways differentiating MD resistant and susceptible chickens, our ability to annotate some immune cell types (especially T cell subsets) was limited by sequencing constraints and further sequencing of more cell types and higher depth would be expected to increase resolution.

In this study we extended this previous analysis^32^ by adding scRNA seq of a first-generation (F1) 6 × 7 population to characterize ASE in individual immune cell types. The joint analysis of scRNA seq of the resistant L6, susceptible L7 and F1 cross provides a deeper understanding of how genetic variation impacts gene expression variation in immune cell types and identifies regulatory mechanisms playing a key role in MD resistance. This is the first study looking into allele specific expression in response to MDV infection at the single cell level. We provide insights on the advantage and limitations of measuring ASE in an F1 population at the single cell level and discuss future directions to expand this area of research.

## Results

### Single-cell RNA sequencing

Single cell RNA seq was performed on 18 samples from MDV infected and uninfected chickens of MD-resistant L6, MD-susceptible L7, and an F1 hybrid L6xL7 population. The single cell libraries were quality filtered for empty cells^36^, cells with low sequence coverage (< 150 genes), and cells determined to be artifactual libraries generated from two cells (> 2,500 unique molecular identifiers (UMI) and > 1,000 genes). Additional filtering was performed to remove apoptotic cells, identified as cells with more than 40% ribosomal gene expression and more than 25% mitochondrial gene expression, and any remaining blood cells with more than 2 reads mapped to the hemoglobin gene HBA1. All the genes mapped to sex chromosomes W and Z were also excluded from the analysis (994 genes). We retained 52,591 cells after quality filtering: 16,068 cells from the F1, 17,349 from L6, and 19,174 from L7. The retained cells had an average sequencing depth of 2 million reads, a median UMI of 619, and median of 419 genes per cell (Supp. Table 1). Cells with active viral infection were identified for L6 (154 cells) and L7 (245 cells) based on the number of viral transcripts quantified for each cell. Viral transcripts were not found in the F1 cells; however viral DNA was detected at low levels in 4 out of 5 challenged birds from the original F1 cohort using whole genome bisulfite sequencing (WGBS), shown in Supp. Figure 1. Two of the birds (3CNT and 1MDV) with WGBS were included in this scRNAseq analysis (Supp. Table 2b).

### Immune cell maps

**Fig. 1:**
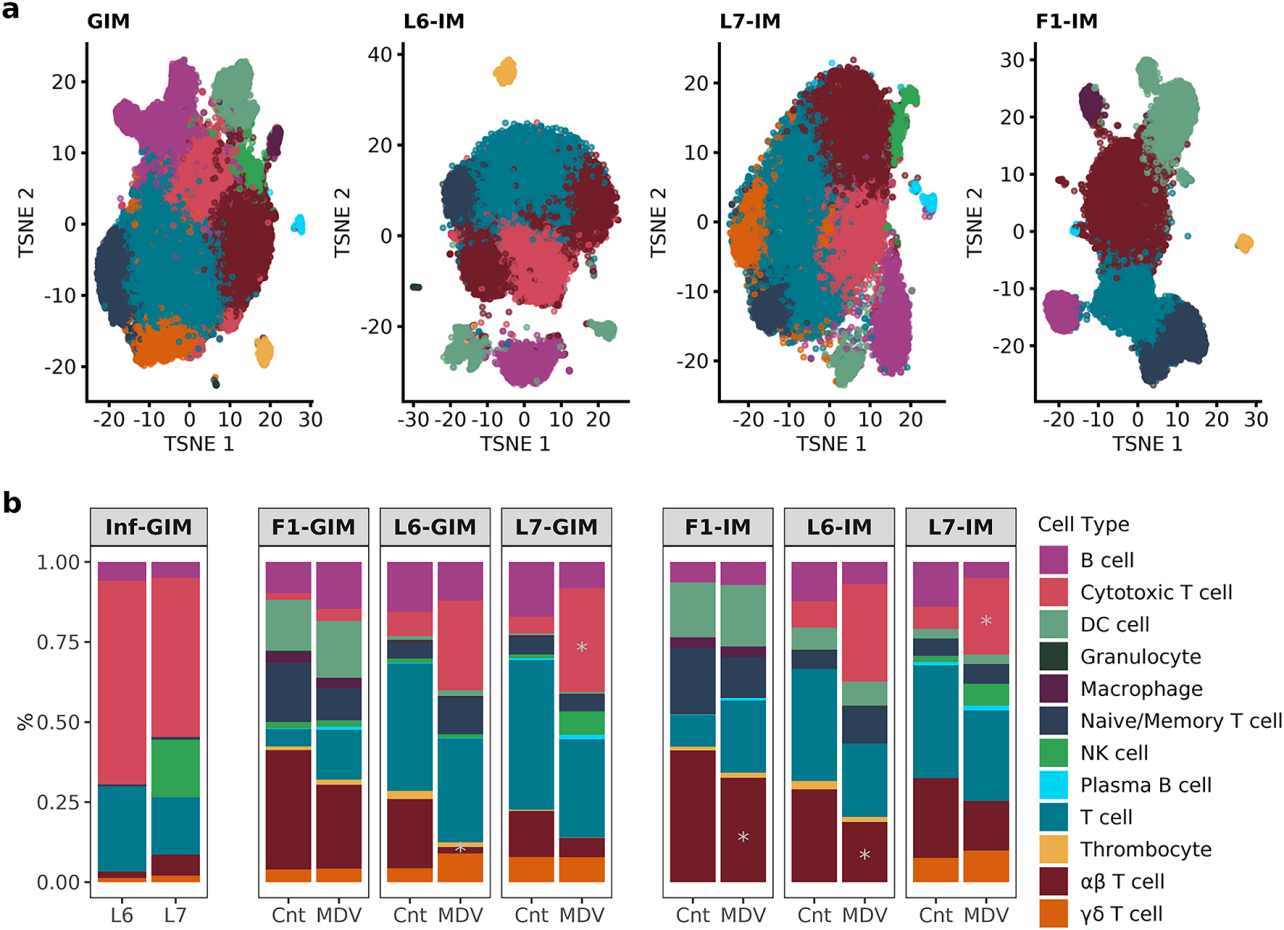
Global and line specific immune cell map. (**a**) TSNE plot depicting a two-dimensional map of immune cell clusters including L6, L7, and F1 in the GIM and per line in the LIM. The clusters from each cell map were annotated to specific cell-types each represented by a different color. (**b**) The proportion of cell types containing MD viral transcripts are depicted in the stacked bar graph color-coded by cell type (Inf-GIM) followed by the proportion of cells in control uninfected (Cnt) and MDV infected samples for the GIM and IM by line. Cell types with significant shifts in the proportion of cells with infection contain a gray asterisk (t-test, pvalue < 0.05).

**Fig. 2:**
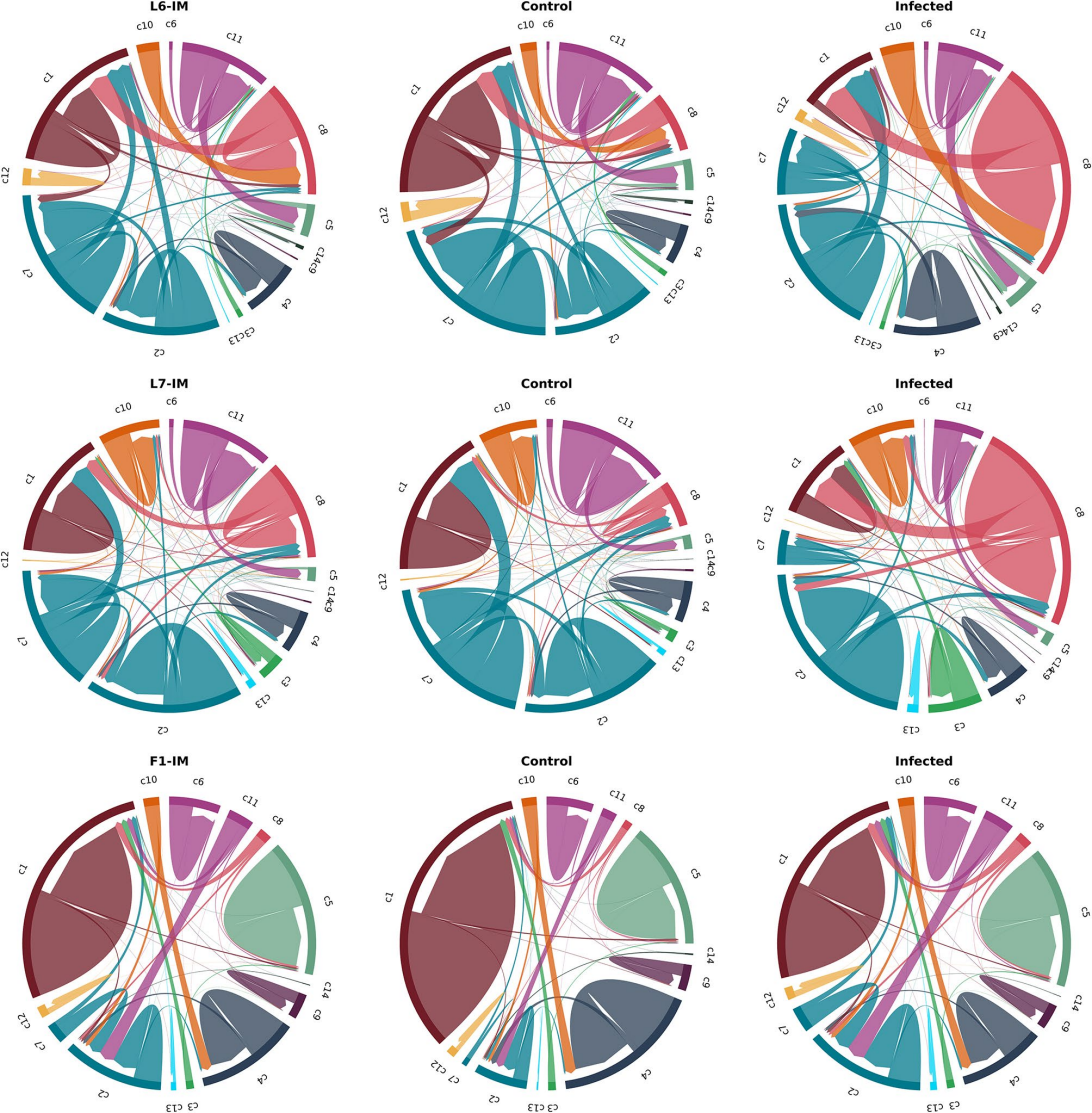
Correspondence between immune cell types identified with the GIM compared to the LIM. Each color corresponds to the cluster in the GIM and the lines within a circle plot represents cells within the cluster and their new location in the LIM. The rows contain circular plots for each experimental line with the 1st column including all cells and the subsequent columns showing the cells from the control uninfected (2nd column) and infected (3rd column) samples.

**Fig. 3:**
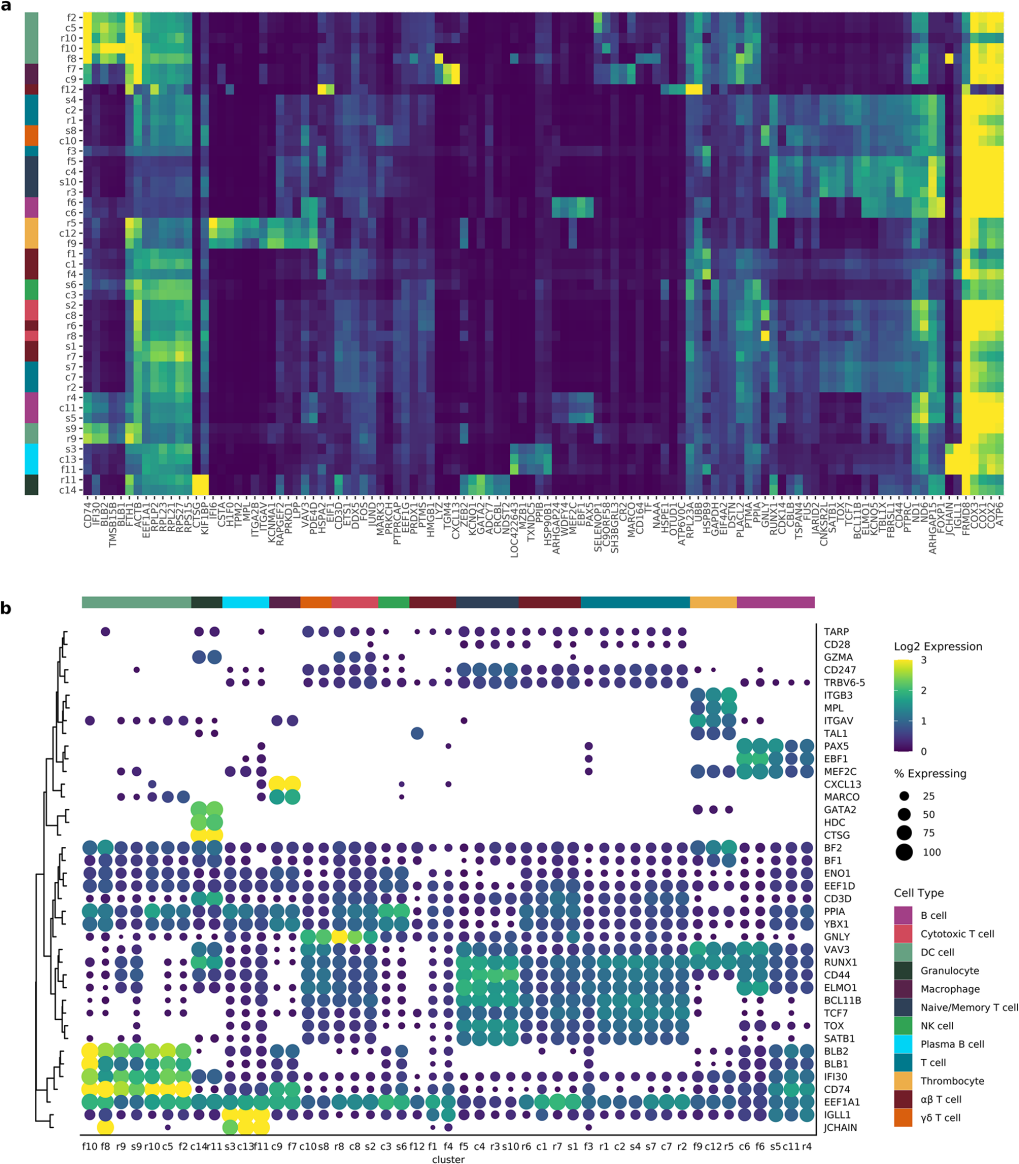
Highest-expressed genes and known immune cell markers used to identify clusters within immune cell maps. Clusters were annotated to immune cell types based on the most highly expressed genes within a cluster supplemented with expression levels of known immune cell type gene markers. (**a**) Heat map of the top 5 expressed genes for each cluster of an immune cell map. The x-axis contains the cluster ID’s (c = GIM, r = L6-IM, s = L7-IM, and f = F1-IM) color coded by cell type and the y-axis the gene names. (**b**) The dot plot shows the percent of cells expressing the selected gene, represented in the size of the dots, and the log2 average count shown with different hues from dark purple (lowly expressed) to yellow (highly expressed) in the color of each dot.

We performed a 2-tier approach to identify and annotate individual immune cell clusters. The 1st tier consisted of a joint analysis, including all samples from the three bird lines, to create a collective immune cell map which we will refer to as the Global Immune Map (GIM) (Fig. 1, Supp. Figure 2, and Supp. Table 2). The 2nd tier separated the cells from each line to create line specific immune maps (Fig. 1, Supp. Figure 2, and Supp. Table 3), where each analysis included only the birds from the line in question (L6, L7, and F1(L6xL7 cross)). We will refer to the line specific analysis as the Line Immune Maps (LIM), one for each line: L6-IM, L7-IM, and F1-IM. The GIM identified 14 cell populations or clusters (c1-14), while the LIM identified 11 clusters for the MD resistant L6-IM (r1-11), 10 for the MD susceptible L7-IM (s1-10), and 12 for the F1-IM (f1-12). We also evaluated the correspondence between the GIM and LIM to ensure the cluster annotations were consistent across line and evaluated subpopulations potentially masked from the GIM (Fig. 2, Supp. Figure 2, and Supp. Table 3). Clusters were annotated to immune cell types based on the top expressed genes for that cluster and the expression of cell type specific gene markers (Fig. 3, Supp. Figures 3–10, Supp. Table 4).

### Innate immune cell types

Avian immunity is a highly regulated system of complex interactions between innate and adaptive immune responses and specialized immune cells that can respond swiftly to invading pathogens. The innate immune response, which include granulocytes, thrombocytes, macrophages, natural killer cells and dendritic cells, as well as innate immune functions in non-immune cell types, are the first line of defense against invading pathogens^37^. Thrombocytes (c12 GIM, r5 and f9 LIM) were identified by the expression of their surface markers thrombopoietin receptor (MPL), integrin beta-3 (ITGB3), and integrin alpha-V (ITGAV), and the expression of the T cell acute leukemia protein 1 (TAL1) transcription factor (Fig. 3). These cells also showed an increased expression of MHC type I molecules (BF1 and BF2). Avian granulocytes are composed of heterophils (the counterpart of mammalian neutrophils), eosinophils, and basophils^37^. The granulocyte population in this study (c14 GIM and r11 L6-IM) was the smallest population (Fig. 1), making up < 1% of the captured cells (this was expected as gradient purification over Histopaque-1077 depleted granulocytes). These cells expressed high levels of the mast cell protease 1 A (CTSG), the transcription factor GATA2, and the enzyme histidine decarboxylase (HDC). The macrophage populations (c9 GIM and f7 F1-LM) were identified by the high expression of their pattern recognition receptor MARCO, the MHC class II antigen gamma chain CD74 and the chemokine CXCL13 also known as B cell attracting chemokine 1. Macrophages and dendritic cells are two types of antigen presenting cells expressing MHC molecules; however, macrophages possess far fewer MHC receptors than dendritic (DC) cells^37^. We were able to distinguish DCs from other antigen presenting cells based on the high expression of MHC class II molecules (BLB1 and BLB2), CD74, the actin-monomer binding protein thymosin beta-15B (TMSB15B), and gamma-interferon-inducible lysosomal thiol reductase (IFI30) involved in antigen processing. The DC clusters were c5 GIM, and r9-10, s9, f2, f8, and f10 from LIM. A smaller cluster appeared intermediate between DCs and NK-like cells (c3 and s6) with a transcription-high signature due to overabundance of ribosomal subunit transcripts. This cluster was classified as NK-like due to expression of granulysin (GNLY) in the GIM, but may be a mixed phenotype (Fig. 3 and Supp. Figures 3–10).

### Adaptive immune cell-types

Lymphocytic cells encompass B and T cell subsets and cytotoxic T and natural killer (NK) cells (although NK cells behave in an innate rather than adaptive manner). The B and T cell subsets can be broadly categorized into functional states with naïve, memory and effector phenotypes (Fig. 3, Supp. Figures 3–10, Supp. Table 4). T cells were further divided into two distinct subpopulations based on their T cell receptor (TCR) class composed of αβ TCR and γδ TCR which are mutually exclusive. The master regulators of the B cell lineage are early B cell factor 1 (EBF1), paired box protein (PAX5), and myocyte-specific enhancer factor 2 C (MEF2C), which were preferentially expressed in c6 and c11 GIM, and r4, s5, and f6 in the LIM. A specialized subset of B cells (c13 GIM, s3 and f11 LIM) was identified as plasma B cells for their high levels of immunoglobulin J chain (JCHAIN) and lambda-like polypeptide 1 (IGLL1, the chicken light chain gene).

T cells were identified by the expression of the T cell master regulator (TCF7), T cell receptor CD3, and zinc finger transcription factor BCL11B (GIM: c1-2, c4, c7-8, and c10; L6-IM: r1, r3, r6, and r8; L7-IM: s1, s2, s4, s7, s8, and s10; F1-IM: f1, f3-5, and f12). Naïve or memory T cell subsets (c4, r3, s10, and f5) were identified as T cell clusters with stable expression of the T cell transcriptional regulators TCF7, BCL11B, TOX and SATB1. In contrast, the effector T cell subsets (c8, c10, r8, and s2) had overall lower expression of these four genes in a reduced number of cells. Cytotoxic T cells (c8, r8, and s2) contained high levels of granulysin (GNLY), granzyme A (GZMA), eukaryotic translation elongation factor 1 alpha 1(EEF1A1) and peptidylprolyl isomerase A (PPIA). The γδ enriched T cell subset (c10 and s8) expressed the T cell receptor gamma protein (TARP) at a higher percent than the other T cell subset while the αβ enriched T cells (c1, r7, s1, f1, f4, and f12) expressed the beta chain (TRBV6-5; Fig. 3).

### Proportional shifts in immune cell populations with infection

The bulk of the cells captured in both the pure lines and F1 belonged to T cell subsets averaging 70%. Differences were observed between the lines when it came to these T cell subsets (Figs. 1 and 2, and Supp. Figure 2). For instance, in the GIM most of the F1 T cells belonged to the αβ enriched T cell subset (c1) accounting for 29% of the F1 cells. In comparison,  within the pure lines the αβ enriched T cell subsets were found at a lower percent (14% in L6 and 12% in L7) in the GIM. Most of the T cells in the pure lines were found in the bulk T cell clusters (c2 and c7) accounting for 37% of the L6 cells and 42% of L7 (Supp. Table 2). The bulk T cell clusters in the GIM showed a variable T cell signature that made it difficult to categorize into a specific T cell subtype. It was evident in the LIM that a portion of cells from the bulk T cell cluster in the GIM were more related to αβ enriched T cells, particularly in the pure lines (Fig. 2, Supp. Figure 2, and Supp. Table 3). We observed an increase in the proportion of αβ enriched T cells in the LIM for all three lines (25% in L6, 22% in L7, and 35% in F1). Based on the LIM, the αβ T cells and cytotoxic T cells subsets were the most responsive to infection (Fig. 1 and Supp. Table 3). A significant reduction of αβ T cells was observed in the F1-IM and L6-IM with the proportion in these clusters dropping by 10% with infection. T cell activation with infection was evident in the pure lines with a significant proliferation of cytotoxic T cells (increasing by 30% in L6 and 24% in L7) concurrent with a downregulation of the T cell subtype (r2 and s4) that fell below 10%.

### Immune cell subpopulations

One aim of this study was to improve the annotation of our prior immune cell map by adding scRNA seq from an F1 cross of the original population. We constructed a GIM to identify cell types shared across the three lines and LIM to evaluate immune cell-subtypes potentially masked from the joint analysis. Evaluating the trajectory of each cell across the different analysis provides a deeper understanding of the heterogeneity within immune cell populations and assists the identification of immune subtypes within each line (LIM) and where they intersect in the GIM (Fig. 2). Overall, the cells in the GIM tended to cluster together into their respective immune cell types in the LIM with a few exceptions. The major differences were observed in the T cell populations where groups of cells shifted from one T cell cluster to another within a specific line (rows of Fig. 2). The LIM showed that groups of cells mapped to T cells and cytotoxic T cells were all clustered together with the αβ enriched T cells in the GIM for all three lines. The majority of the γδ enriched T cells from L6 in the GIM were grouped with the cytotoxic T cells in the L6-IM while in the F1-IM these cells were clustered with the naïve/memory T cells. Contrary to L6 and F1, the γδ enriched T cells in L7 stayed within its cluster in the L7-IM. The only other cell type that showed a compositional shift between the GIM and LIM was a subset of B cells. In the L6-IM a group of cells from the GIM c11 B cell cluster were reclassified as DCs in the L6-IM. These cells did not express the classical B cell markers EBF1 and PAX5 and showed increased expression of the interferon gamma inducible lysosomal thiol reductase IFI30 and the zinc finger E-box binding protein ZEB2 (Supp. Figures 5–6). IFI30 is constitutively expressed in antigen presenting cells and plays a role in MHC class II antigen presentation while ZEB2 has been shown to be important for the development of plasmacytoid dendritic ^38^ (pDC) cells. The lack of interferon expression in these cells, a hallmark of pDCs, along with the increased expression of MHC type II molecules, IFI30, and ZEB2 led us to the general definition of DCs for this population. In the F1-IM a subset of the c11 B cell cluster was grouped together with T cells (3rd row Fig. 2). A closer look at the transcriptional signatures from this cluster showed an increased expression of ribosomal genes and metabolic genes such as NADH dehydrogenase subunits and cytochrome b in the T cell cluster c2 and B cell cluster c11 from the GIM and the T cell cluster f3 from the F1-IM (Supp. Table 3). These signatures are consistent with a proliferation state and clonal expansion of activated lymphocytes, a canonical signature shared between B and T cells^39^. Consequently, it is not surprising that these cells were grouped together based on their cellular state rather than cell type in the GIM. As demonstrated in recent single cell studies^39^, NADH signatures are predictive of TCR driven T cell expansion which was observed in this F1 T cell population with infection. These compositional shifts between T and B clusters were observed in control and infected samples, therefore not biased towards a treatment group (columns 2 and 3 in the  3rd row of Fig. 2).

### Differential expression analysis

**Fig. 4:**
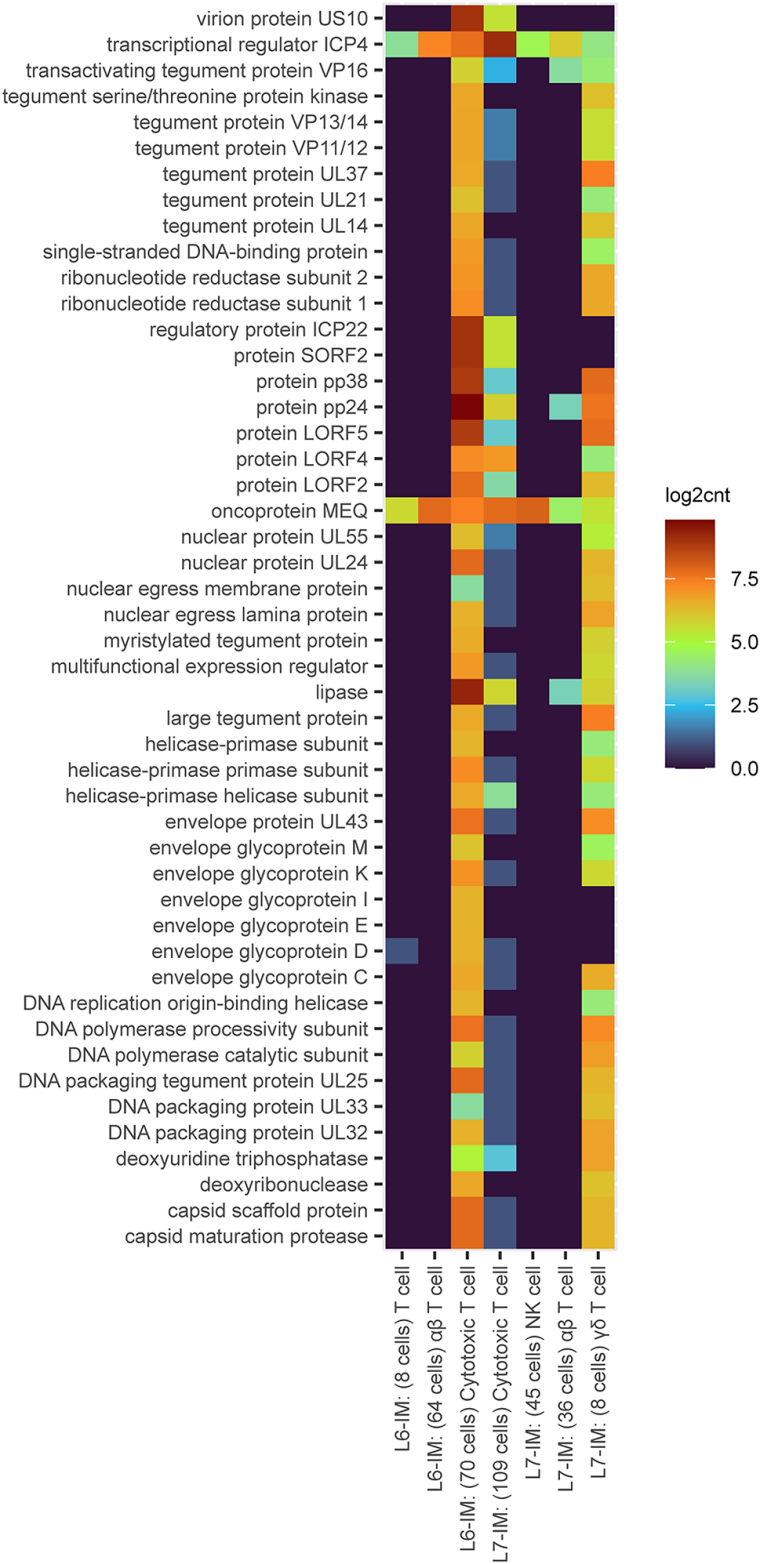
MD viral gene expression. MDV genes were quantified in a small number of cell types in L6 and L7. The cell types and number of cells with viral reads are on the x axis and the MDV genes on the y axis. The log2 counts per MDV gene are shown in this heatmap with blue tones depicting low expression and yellow to red higher expression. The MDV oncogene MEQ and early lytic marker ICP4 were expressed across all the cell types with viral transcripts.

We assayed whether immune cells responding to lytic viral replication behaved differently between L6 and L7. This analysis included 399 cells expressing viral MDV transcripts which are observed solely in the pure lines (245 L7 cells and 154 L6 cells) and enriched in cytotoxic T cells (Table 1). Significant differences were observed between L6 and L7 infected cells in the B cells, αβ T cell, and cytotoxic T cell populations. Infected B cells and αβ enriched T cells had altered expression of components of the mitochondrial electron transfer chain that regulate cellular energy metabolism. We observed higher levels of cytochrome c oxidase (COX1 and COX3), and NADH dehydrogenase (ND6) in L6 αβ enriched T cells and a slight increase of cytochrome B (CYTB) in L7 B cells. The major differences were observed in cytotoxic T cell populations and upregulated in L6 compared to L7. The top upregulated genes in L6 mapped to endogenous retrovirus sequences, endogenous retrovirus group K (LOC107052718) and endogenous avian leukosis virus subgroup E (LOC121107448). The other genes with increased expression in L6 were granulysin (GNLY), granzyme M (GZMM), PLAC8 like 2 (PLACL2), and testis expressed protein 14 (TEX14). The only gene upregulated in L7 compared to L6 in cytotoxic T cells was the nucleophosmin/nucleoplasmin 3 (NPM3). NPM3 plays a key role in chromatin remodeling and its heightened expression in the susceptible line suggests that MDV may modulate the expression of this gene in some way. We then looked at the MD viral genes expressed in these infected cells (Fig. 4). The MDV transcriptional regulator ICP4 and oncoprotein MEQ were expressed in all the infected cell types and for some these were the only genes quantified. The cell types with the most prolific MDV signatures were the L6 cytotoxic T cells and L7 γδ T cells. The level of viral transcripts in the F1 cross in this analysis was too low to quantify (Supp. Figure 1).

We performed a differential expression (DE) analysis for the GIM and the LIM excluding the cells with MD viral transcripts which were included in our previous analysis. The GIM analysis identified 194 genes for L6, 797 for L7, and 445 for F1 (Supp. Figure 11 and Supp. Table 5). The cell types most responsive to infection in the GIM were the T cells (c2) for the pure lines and the naïve/memory T cells (c4) for the F1. The LIM identified 205 differentially expressed genes for L6, 2116 for L7, and 319 for F1 (Supp. Figure 11, and Supp. Tables 6–8). Contrary to the GIM, the LIM identified the cytotoxic T cells in L6 (r6), and the αβ T cells in L7 (s1) as the most responsive to infection while the naïve/memory T cells (f5) remained the most responsive for the F1-IM. Notably, 92% of the responding genes in naïve/memory T cells from the F1-IM were significantly downregulated with infection and enriched in biological pathways associated with the regulation of DNA-templated transcription and RNA metabolic process modulating cytokine production and cell differentiation (Supp. Table 8b). The aim of this approach was to evaluate the immune cell landscape from a global perspective as cells with similar molecular signatures will group together in the GIM representing the main features characteristics of that cluster. Meanwhile, the LIM captures the intrinsic signatures that can distinguish potential subpopulations or transient states.

### Allele-specific expression analysis

**Fig. 5:**
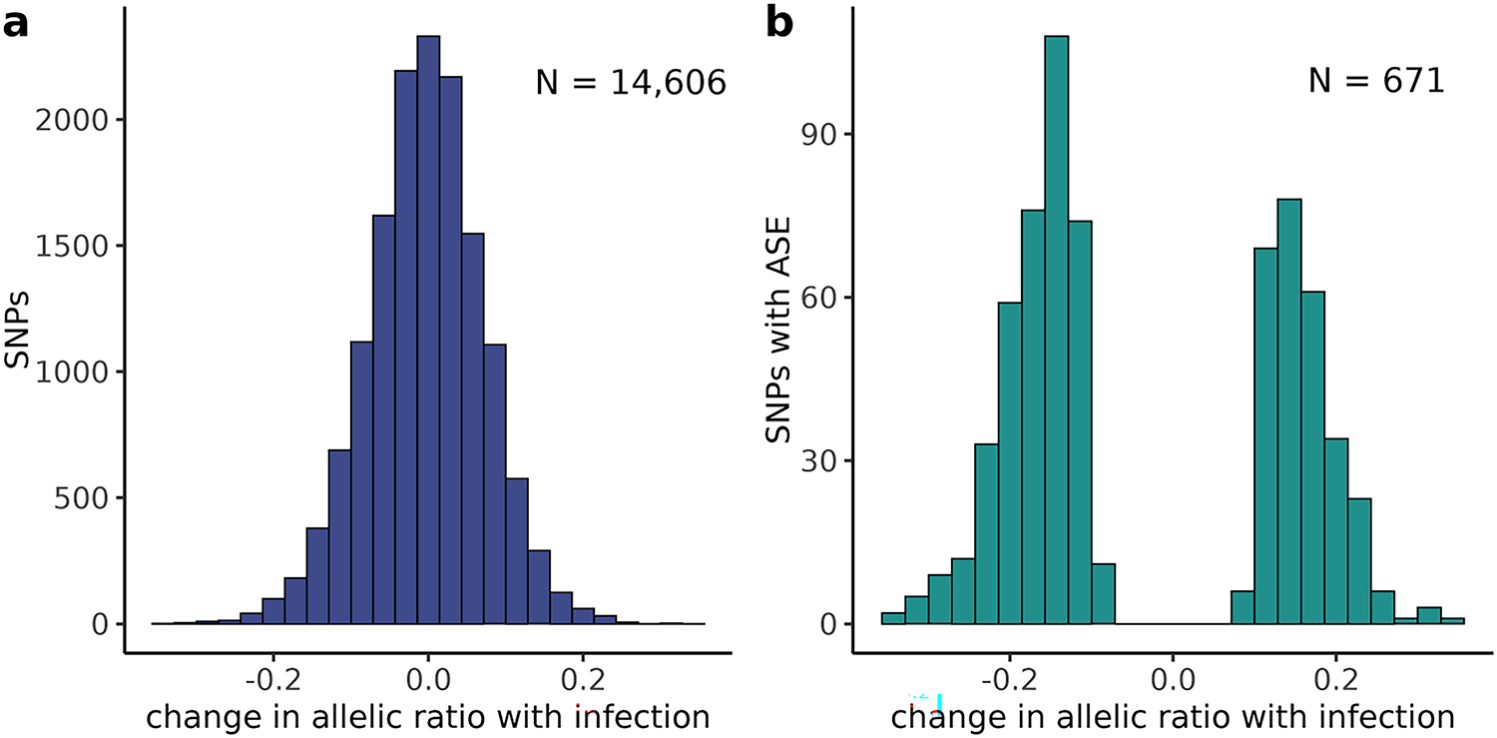
Marek’s Disease induces shifts in the allelic ratio of genomic loci implicated in disease resistance/susceptibility. (**a**) Difference between the allelic ratio (alternative allele count / total allelic count) of infected samples versus controls for all the analyzed SNPs. (**b**) SNPs with ASE showed a shift towards a bimodal distribution in allelic ratios with MDV infection.

**Fig. 6:**
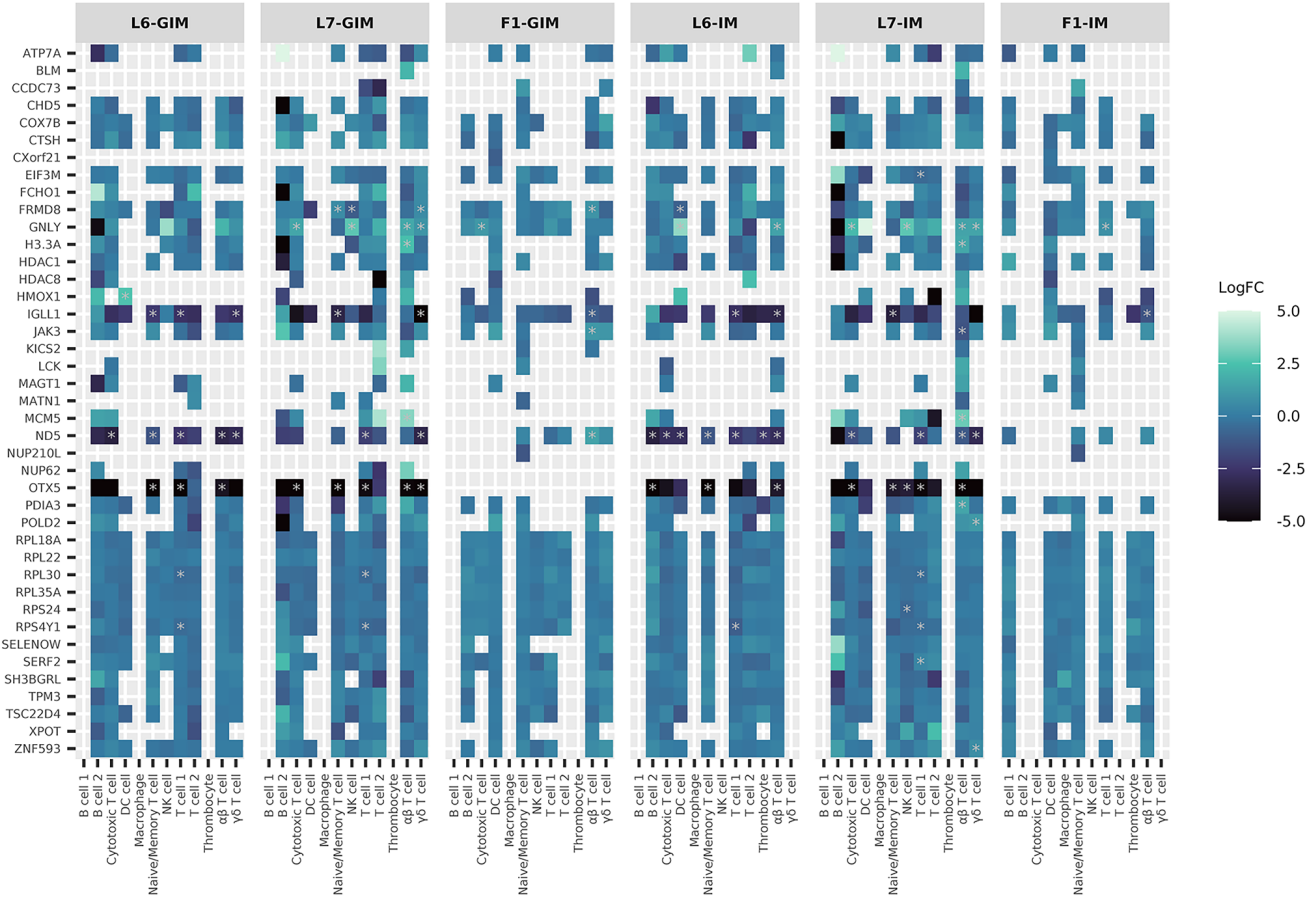
Candidate MD resistant gene expression across cell types. Heatmap of log^2^ FC differences in gene expression with infection across the different immune maps tested. Genes included in this graph were mapped to SNPs exhibiting significant ASE in response to infection. Gray asterisks depict a significant difference in the gene’s expression (y axis) for that cell type (x axis) and immune map.

Single nucleotide polymorphisms (SNPs) were called from the transcriptomes using GATK and their allele specific expression (ASE) quantified as the sum of allelic counts across all cells in a sample. We identified 14,606 SNPs segregating as a heterozygous genotype in all F1 samples with an average allelic count of 40 reads (Supp. Figure 12). The ASE analysis identified 671 SNPs exhibiting ASE in response to infection (average allelic ratio (AR) difference of 0.16 ± 0.05 stdev; p-value < 0.05, Fig. 5, Supp. Table 9). These SNPs were annotated to 839 genes of which 181 were found differentially expressed in specific cell types (Supp. Table 10) with 30% of these enriched for molecular functions associated with nucleic acid bind (PPI enrichment p-value 2.83e-05, Supp. Figure 13). The next step was to test if ASE was unique to an immune cell type. For this analysis we retained 259 SNPs with allelic expression across all samples to be able to define from which parental line the alternative allele was inherited. Of the 259 SNPs, 22 showed significant allelic bias in response to infection (average AR difference of 0.27; p.value < 0.05) in 8 cell types (Table 2). These 22 SNPs were annotated to 59 genes at an average distance of 1.24 Kb from the transcription start site (Supp. Table 10). More than 50% of these genes were also differentially expressed in the DE analysis (Fig. 6).

## Discussion

MD genetic resistance has been attributed to molecular mechanisms controlling the transcriptional response to MD viral infection using bulk RNA sequencing of immune cells and targeted sequencing of CD4 T cells^22,23^. In this study we showed that 30% of genes mapped to ASE SNPs and differentially expressed in immune cell types in response to infection are associated with transcription activation and nucleosome organization pathways. While our ability to measure ASE at the single cell level was limited due to the low depth of coverage in F1, we were able to capture ASE signatures in T cell subsets, DCs and macrophages. We successfully quantified MDV viral genes at the single cell level and show that the early lytic phase gene ICP4 and the oncogene MEQ are expressed in all infected immune cell types. The addition of the F1 line greatly assisted in the annotation of T cell subtypes and in the detection of additional immune cell types and states that enhanced our understanding of chicken immune cell classifications.

In this study we evaluated host immune response to MDV infection 6 days post infection corresponding to the early cytolytic phase of MD where the virus is actively replicating inside infected cells (2–7 dpi; peaks at 4dpi) and before the latent phase (7–10 dpi)^40^. To characterize the immune cell microenvironment when a cell is activated to control an internal infection, we focused our analysis on actively infected cells from L6 and L7. MDV viral transcripts were found enriched in cytotoxic T cells for both L6 and L7. The resistant L6 response to infection was stronger than L7, in both actively infected cells and cells responding to the infection. We observed increased expansion of cytotoxic T cells and higher levels of GNLY and GZMM, as seen in our previous analysis^32^. In contrast we found increased expression of two mapped endogenous retrovirus sequences in L6 infected cytotoxic T cells, endogenous retrovirus group K (LOC107052718) and endogenous avian leukosis virus subgroup E envelope gene (mapping to LOC121107448 in the broiler reference). Endogenous retroviral (ERV) sequences are the remnants of previous retrovirus (RNA viruses) infection that have integrated into the host genome leaving a fingerprint of retroviral lineages that make up roughly 3% of the avian genome^41^. ERVs are frequently found to produce transcripts in various chicken tissues, some with detrimental effects on productions traits, and while little is known as to why or how these elements are induced, altered expression of these elements can occur with MDV infection, as has been shown for the endogenous Avian Leukosis Virus subgroup E 1 (ALVE1)^42,43^. ALVE1 is known to segregate in L7 and ALVE3 in L6^8^, however ALVE1 and ALVE3 share high sequence similarity but differ in insertion site, as well as ALVE genome integrity^44^, so differences in basal expression of ALVE genes and potentially differential expression are to be expected, but further research is needed to elucidate any functional impact on MDV replication.

Infected B cells and αβ enriched T cells had altered expression of components within the mitochondrial electron transfer chain that regulate cellular energy metabolism through oxidative phosphorylation. L6 αβ enriched T cells showed higher levels of cytochrome c oxidase (COX1 and COX3), and NADH dehydrogenase (ND6) and L7 B cells had reduced expression of cytochrome B (CYTB). Activated immune cells undergo extensive metabolic reprogramming to support specific immune functions^45^; conversely viruses have evolved strategies to highjack hosts’ cellular metabolism to support their own viral replication and limit immune activation^46^. We do not know which of these processes are driving the observed differences or perhaps it is an interplay of both since MDV viral replication was evident in these cells. More research is needed to investigate the effects of immunometabolism on MDV viral replication efficiency.

Thrombocytes play an important role in the innate immune response. Pathogen recognition receptors are numerous on the cell surface of thrombocytes and when activated can release antimicrobial proteins to limit the spread of infection and immune signaling proteins to initiate an inflammatory response and recruit immune cells to the area of infection^37,47^. With infection, L6 thrombocytes reduced the expression of their MHC II receptors (CD74 and BLB1) and decreased the expression of a core subunit of the mitochondrial respiratory chain complex 1 (MT-ND1). The reduced expression of MHC receptors may indicate a transition to a more activated state, however, more research on the role thrombocytes play in MDV immunity is needed as they have not been studied in this context. Thrombocytes may play a bigger role than is currently known and may influence the progression of MDV and development of tumors^48^.

The naïve/memory T cell population was the most responsive to infection in the F1 population. Several key transcriptional modulators of T cell activation were significantly downregulated in this cell type including interferon regulatory factor 4 (IRF4), activating transcription factor 3 (ATF3), nuclear receptor subfamily 4 group A member 1 (NR4A1), and a zinc finger cofactor of the GATA1 transcription factor (ZFPM1). It is worth noting that none of these transcription factors were found differentially expressed in the pure lines indicating this signature might be a unique response to MDV in F1. IRF4 and ZFPM1 are important regulators of the helper T cell response. IRF4 expression is positively correlated with the strength of T cell receptor signaling and deficient expression of IRF4 can lead to impaired helper T cell differentiation and limited proliferation^49^. ATF3 is a stress response protein that can enhance the accumulation of the herpes simplex virus 1 (HSV-1) latency-associated transcript (LAT) in infected B cells and is implicated in HSV-1 reactivation^50^. The top two upregulated genes in F1 naïve/memory T cells were vascular endothelial growth factor C (VEGFC) and a tumor necrosis factor receptor-associated factor (TRAF) family member associated NF-κB activator (TANK). Interestingly, TANK protein is a known inhibitor of Epstein-Barr virus oncoprotein-mediated activation of NF-κB by blocking viral latent membrane protein 1 (LMP1) interactions with TRAF^51^ which can hinder the development of B-cell lymphomas^52^. VEGFC expression promotes the infiltration of naïve and regulatory T cells, a response that has been used to induce enrichment of tumor-infiltrating naïve T cell in cancer immunotherapies. For instance, the cancer vaccine VEGFC-vax overexpresses VEGFC and when administered locally can promote an antitumor T cell response^53^. The establishment of latency occurs at approximately 7 dpi in the MDV life cycle and during this period viral gene expression is limited to MDV latency-related genes^54^. Therefore, the gene signatures observed in F1 naïve/memory T cells may reflect an early host response to MDV genome integration rather than lytic replication which could explain in part the reduced expression of T cell activation markers.

Differences between the global immune map and the line specific maps showed that T cell subtypes regrouped into clusters with similar effector functions. For instance, in the L6-IM the cytotoxic T cells separated into two groups, with each one containing γδ T cells, bulk T cells and cytotoxic T cells from the GIM. These two effector T cell populations contained the bulk of infected cells in L6 which was only 3% of cells. Interestingly, only one of these cytotoxic T cells exhibited an activated phenotype with an increased expression of cytokine receptors including interleukin-2, 6, and 8 receptors (IL2RA, IL6ST, and IL18R1), C-C motif chemokine receptor 5 (CCR5), and interferon gamma receptor 1 (IFNGR1) as well as increased antigen processing and presentation through the MHC class I pathway (TAP1, TAP2, BF2, and AP3B1), and autophagy, based on increased phosphatidylinositol 3-kinase activity (PIK3C3). PIK3C3 plays a critical role in autophagosome nucleation, a process that captures and degrades cytoplasmic components, and PIK3C3-deficient mice showed impaired T cell receptor-induced proliferation and was suspected to cause dynamic changes in effector T cells, NK cells and B cells^55^.

ASE screens are a valuable tool for identifying functional variation implicated in genetic resistance to MD. Biallelic SNPs exhibiting allele specific expression in response to infection could be functionally linked to causative polymorphisms that alter the transcriptional regulation of their associated genes. In this study we evaluated ASE within each immune cell type identified in our F1 population. Several SNPs exhibiting ASE in specific immune cell types mapped to genomic regions containing genes with critical functions regulating translation including ribosomal subunits and translation initiation factors, as well as genes regulating genome organization and structure like histones and histone deacetylase. If the same ASE signature was captured in more than one cell it showed a conserved pattern of expression. One of these ASE SNPs identified mapped to the transcriptional genes KICS2, SNORA86 (small nuclear RNA gene), XPOT (nuclear export receptor for TRNA), and RPL18A (60 S ribosomal protein L18a). This SNP showed the same biased expression towards the L7 allele with infection in two T cell clusters, the F1 naïve/memory T cells (f6) and T cell subset (f3). KICS2, also known as C14orf166, has been implicated to play an important role in RNA metabolism, centrosome structure, and viral infection^56^. Specifically, KICS2 has been shown to interact with influenza A virus proteins as well as hepatitis C virus proteins to promote viral infection. In dendritic cells, a mini-chromosome maintenance protein (MCM5) and the critical stress response gene, heme-oxygenase 1 (HMOX1), were mapped to a SNP with a biased expression towards the L7 allele. Infection reduced the expression of the L7 allele. MCM5 protein, known to maintain the formation of the replicative fork structure during DNA replication and the reparation of double stranded breaks, also assists the viral replication of HIV-1^57^, Epstein-Barr virus^58^, and influenza type A virus^59^. Moreover, studies on SARS-CoV2 have shown HMOX1 can potentially block viral replication of this virus in several cell lines^60^. Ribosomal proteins RPS4Y1, RPS24, and the translation factor EIF3M are targets for viral-host protein interactions in *in vitro* studies of H5N1 influenza A virus^61^. Therefore, L7 susceptibility to MD may be related to defects in the regulation of DNA maintenance and repair mechanisms which are exploited by MDV to increase viral replication. However, SNPs with ASE were also found in surrounding genes with critical functions in immune response including granulysin (GNLY), a component of the MHC class I peptide loading complex PDIA3, immunoglobulin lambda-like polypeptide 1 (IGLL1), lymphocyte-specific protein tyrosine kinase (LCK), and janus kinase 3 (JAK3). Altogether, these results show that indeed MD genetic resistance is strongly influenced by molecular mechanisms controlling the transcriptional response to MD viral infection.

The expression of CD4 and CD8 receptor genes in the T cell subsets in the F1 was low and captured in a small percent of cells, like in our previous study^32^. We were still unable to distinguish between CD4 and CD8 T cells. However, we were able to capture some of the ASE signatures measured directly in CD4 T cells using the same population used in this study^23^. Consistent with this study, we observed ASE in SNPs mapped to SLC43A2, PPFIA1, and CBLB, but only in the bulk ASE analysis. We suspect this is due to the low depth of coverage in the F1 and low CD4 gene expression that made it difficult to parse CD4 T cells. In future studies by using CD4 and CD8 monoclonal antibody tagged with unique sequence barcode tags^62^, we can distinguish between CD4 and CD8 T cells by using the antibody specific sequence as a proxy to quantify CD4 protein surface expression. This would greatly assist in the further characterization of these immune cell subtypes.

In this study we used three genetically diverse chicken lines to study the distinct roles different immune cells play when responding to a Marek’s Disease viral infection. We were able to identify immune cell populations that had not previously been described in the chicken based on their gene expression signatures and MDV viral gene expression. Different strategies were investigated to better characterize avian immune cell types with the addition of the F1 population proven to be useful to parse transient states in subpopulations of cells. However, further research is needed to improve our characterization of these immune cell types. The major finding of this study was that genetic resistance to MD is strongly influenced by molecular mechanisms controlling the transcriptional response to MD viral infection. We were able to capture ASE signatures within specific immune cell types and found that genomic regions with ASE SNPs, for the most part, contained more than one gene of critical importance for transcriptional regulation.

## Methods

### MDV challenge and sample collection

The experimental work presented in this research was approved and carried out in accordance with the ARRIVE guidelines and the Institutional Animal Care and Use Committee, USDA, ARS, ADOL, East Lansing, MI (IACUC approval #2022-08). The experimental design consisted of 20 ADOL line 6_3_ × 7_2_ F1 chickens randomly allocated to either an MDV-challenge or control (unchallenged) group, i.e., 10 chicks per condition to satisfy welfare requirements per Horsfall-Bauer (HB) isolation unit. The challenged group received an intra-abdominal injection at two weeks of age with 2,000 pfu JM/102W strain MDV. Birds were housed 6 days post-infection in HB units and humanly euthanized individually using CO_2_ exposure following the poultry standard operation procedures stipulated in the AVMA Guidelines for the Euthanasia of Animals. Whole spleens were subsequently collected for each bird, and splenic-derived leukocytes were isolated. Briefly, individual spleens were homogenized and filtered through a 40µM cell strainer to obtain single-cell suspensions. Mononuclear leukocytes were enriched with a density gradient cell separation protocol using Histopaque-1077 medium (MilliporeSigma, Burlington, MA). Six single-cell suspensions (2 × 10^5^ cells from 3 control and 3 MDV-infected samples) were transferred the same day on ice to the Michigan State University Research Technology and Support Facility Genomics Core for 10X Genomics single-cell RNA sequencing. DNA was isolated from the splenic-derived lymphocytes of 10 F1 birds (5 per condition) using the QIAamp DNA Mini Kit (Qiagen Cat. No. 51304) following the manufacturer’s protocol and sent to LC Sciences (Huston, TX) for whole genome bisulfite sequencing (WGBS, data not shown). The pure lines 6_3_ and 7_2_ MDV-challenge study was described previously^32^.

### 10X Genomics sequencing

Chromium Next GEM Single Cell 3’ kit v3.1 was used to prepare the single-cell libraries on the 10X Genomics platform (10X Genomics Inc., Pleasonton, CA) with a target number of 10k cells per sample. The 10X single-cell libraries were sequenced on the NovaSeq 6000 system (paired-end 28 bp x 90 bp) (Illumina, San Diego, CA). Raw Illumina sequences were demultiplexed and converted to single cell feature counts using CellRanger v7.0.0 (10X Genomics) and mapped to an amalgamated reference genome that included the reference Gallus gallus (GCF016699485.2) and Gallid alphaherpesvirus 2 (GCF000846265.1). Viral gene expression was quantified for each cell containing reads mapped to the MDV genome by counting the number of reads that uniquely aligned with known MDV gene positions using SAMtools bedcov v1.19.2^63^. We used WGBS to quantify viral DNA in the F1 birds. Briefly, DNA sequences were quality trimmed to remove adapter sequences, the first 6 leading bases and 3 trailing bases per read using Trimmomatic v0.39-Java-11^64^. The quality filtered DNA reads were mapped to the amalgamated chicken and MD virus genome using BWA-meth v0.2.5^65^ and reads mapped to the viral genome quantified using samtools coverage v1.19.2.

### Single-cell data analysis

Data analysis was performed in R following procedures described in Orchestrating Single-cell Analysis in Bioconductor^66^. Quality filtering included the removal of droplets that failed to capture an individual cell (DropletUtils^36^), cells with low numbers of unique molecular identifiers (UMI) or total read counts (scater^67^). Additional filtering of blood cells and cells with a high percent of mitochondrial or ribosomal gene expression were also removed. The batch-correcting mutual nearest neighbor (MNN) algorithm^68^ was used for data integration and normalization of the F1 and pure lines 6 and 7 single-cell experiments. The Euclidean distances across the top 1,000 genes with heterogenous expression across cells were used to reduce the dimensionality of the single-cell data with principal component analysis and cell clusters identified with t-distributed stochastic neighbor embedding (t-SNE) and Louvain multi-level modularity optimization algorithm to connect each cell to its nearest neighbors in the high-dimensional space. A two-tier analysis was implemented to identify the cell populations found across all samples, creating a global immune map (GIM), and from cell populations enriched within an experimental line, to create a line specific immune map (LIM). Clusters were annotated based on the expression profiles of the topmost upregulated genes within a cluster and known molecular markers specific to cell types or biological states. We compared the GIM to the LIM using circular correspondence graphs where each cell in the LIM is mapped back to the GIM creating a trace path to potential subpopulations of cells. To determine the molecular profiles of responding cell types, a pseudo-bulk differential expression analysis was performed, where each gene is represented as an aggregated expression for all the cells in a cluster. The limma empirical Bayes analysis with voom precision weights was used to determine significant differentially expressed genes responding to infection. We assessed pairwise comparisons within and across populations for each cell type identified. Functional enrichment analysis was performed for genes with significant variable expression in an immune cell type. We used the Gene Ontology, KEGG pathways, and TRANSFAC regulatory motif matches databases in the enrichment analysis^69^.

### SNP detection and allele-specific expression

The mapped transcripts for all quality-filtered cells were used to call single nucleotide polymorphisms (SNPs) following GATK (Broad Institute, Cambridge, MA) best practice workflows for identifying short variants in RNAseq data with allele-specific annotations. GATK obtains a bulk estimation of allelic counts for all cells sequenced per sample. Multiallelic and fixed SNPs were discarded from further analysis. Biallelic SNPs with a genotype coverage of at least 6 reads and genotype call in at least two birds per group were retained for the allele-specific expression (ASE) analysis to identify allelic imbalances caused by MDV infection. We used CellSNP^70^ to pileup single-cell allelic counts for known SNPs previously mapped using GATK. ASE was performed in two ways: a bulk analysis where the allelic counts were collapsed across all cells for a single bird (GATK) and a cell-type specific analysis collapsing allelic counts for each cell cluster per bird. A quasibinomial logistic regression with overdispersion was used to estimate the allelic ratio of each group (infected and uninfected) followed by a Wald chi2 to test the null hypothesis of no difference in allelic expression with infection.

**Table 1:** Genes differentially expressed in infected cells from line 7 compared to line 6.

Gene	Cluster	Cell type	Log_2_FC	Log_2_CPM	pvalue	FDR
COX1	1	αβ T cell	-1.87	12.93	4.85E-04	3.64E-03
COX3	1	αβ T cell	-1.54	13.01	2.14E-03	1.07E-02
ND6	1	αβ T cell	-3.10	11.72	2.81E-04	3.64E-03
LOC107052718	8	Cytotoxic T	-4.91	7.49	2.58E-06	1.44E-03
LOC121107448	8	Cytotoxic T	-5.58	8.00	4.51E-12	5.02E-09
PLACL2	8	Cytotoxic T	-0.86	11.48	1.75E-04	4.88E-02
NPM3	8	Cytotoxic T	2.14	7.81	3.08E-04	4.90E-02
TEX14	8	Cytotoxic T	-1.47	8.61	2.20E-04	4.89E-02
GNLY	8	Cytotoxic T	-0.64	13.41	3.02E-04	4.90E-02
GZMM	8	Cytotoxic T	-1.31	9.39	6.89E-05	2.56E-02
CYTB	11	B cell	0.67	12.88	2.97E-03	2.97E-02

**Table 2:** SNPs exhibiting ASE within an immune cell type in F1 cross.

SNP	Parent	MDV	Cnt	Diff	Chi^2^	*p*-value	Cell Type	Genes
chr1:33731077_T/C	L7	0.58	0.32	0.27	3.87	4.19E-02	T cell	KICS2, SNORA86, PRB1, RPL18A, XPOT
	L7	0.68	0.47	0.21	3.89	4.84E-02	Naive/Memory T	
chr1:52145836_C/T	L7	0.35	0.54	-0.19	4.04	4.43E-02	DC cell	HMOX1, MCM5
chr2:127057343_T/C	L7	0.50	0.40	0.10	3.94	4.72E-02	αβ T cell	ERICH5, SNORA72, MATN2, RPL30
	L7	0.48	0.31	0.17	4.40	3.59E-02	DC cell	
chr4:12863383_C/T	L7	0.49	0.17	0.32	4.76	2.92E-02	αβ T cell	ATP7A, COX7B, MAGT1, TRNAL-CAA
chr4:1750499_C/T	L7	0.60	0.27	0.33	6.82	9.04E-03	T cell	TSC22D4
chr4:1848886_T/C	L6	0.68	0.29	0.39	6.24	1.25E-02	DC cell	HDAC8, NUP62, RBM41, RPS4Y1
chr4:9301316_T/C	L7	0.34	0.63	-0.28	5.16	2.31E-02	DC cell	CXorf21, SH3BGRL
chr5:5734254_A/G	L7	0.42	0.70	-0.27	4.38	3.63E-02	Naive/Memory T	CCDC73, EIF3M
chr6:13931127_A/G	L6	0.44	0.62	-0.18	4.75	2.94E-02	T cell	RPS24
chr9:14822550_C/T	L7	0.52	0.41	0.12	4.87	2.73E-02	Macrophage	IQCG, LOC121111469, RPL35A
chr10:19951451_T/C	L6	0.61	0.24	0.37	6.60	1.02E-02	DC cell	BLM, CTSH
chr10:20158445_A/G	L6	0.47	0.31	0.16	4.69	3.03E-02	DC cell	RASEF, PDIA3, SERF2, SERINC4
chr15:7933337_G/A	L6	0.60	0.45	0.14	5.20	2.25E-02	T cell	IGLL1
chr21:633266_T/C	L6	0.56	0.36	0.20	4.27	3.88E-02	DC cell	CHD5, RNF207, RPL22
chr22:4551631_C/T	L6	0.44	0.68	-0.24	4.44	3.52E-02	αβ T cell	GCK, GNLY, POLD2
chr23:5499769_C/T	L7	0.57	0.17	0.40	4.82	2.81E-02	Naive/Memory T	HDAC1, LCK
chr23:5499819_A/G	L6	0.91	0.38	0.53	4.84	2.77E-02	αβ T cell	HDAC1, LCK
chr23:616164_A/G	L6	0.35	0.59	-0.24	4.11	4.27E-02	αβ T cell	CNKSR1, MATN1, ZNF593
chr25:1874872_A/G	L7	0.49	0.09	0.40	4.43	3.53E-02	T cell	NUP210L, TPM3
chr28:3770449_G/A	L7	0.45	0.80	-0.35	4.41	3.57E-02	Naive/Memory T	B3GNT3, JAK3, FCHO1
chr38:256109_T/C	L6	0.76	0.30	0.46	5.11	2.38E-02	DC cell	H3.3 A, OTX5, SELENOW
chr39:38440_C/A	L6	0.50	0.32	0.18	9.16	1.94E-03	αβ T cell	FRMD8, ND5

## Electronic supplementary material

Below is the link to the electronic supplementary material.

Supplementary Material 1

Supplementary Material 2

## Data Availability

The datasets generated and/or analyzed during the current study are available in the Gene Expression Omnibus (GEO) under GSE202739 for L6 and L7 scRNA seq and GSE278696 for the F1.
