# Supplementary material for: scRNA seq of an F1 cross of Marek’s disease resistant and susceptible chickens identifies allele specific expression signatures enriched in transcription modulators
Source: Sci Rep. 2025 Jan 29;15:3689. doi: 10.1038/s41598-025-86174-w (PMC11779831; doi:10.1038/s41598-025-86174-w)
Supplement: Supplementary file 1 — Supplementary Material 1 [file 41598_2025_86174_MOESM1_ESM.docx]

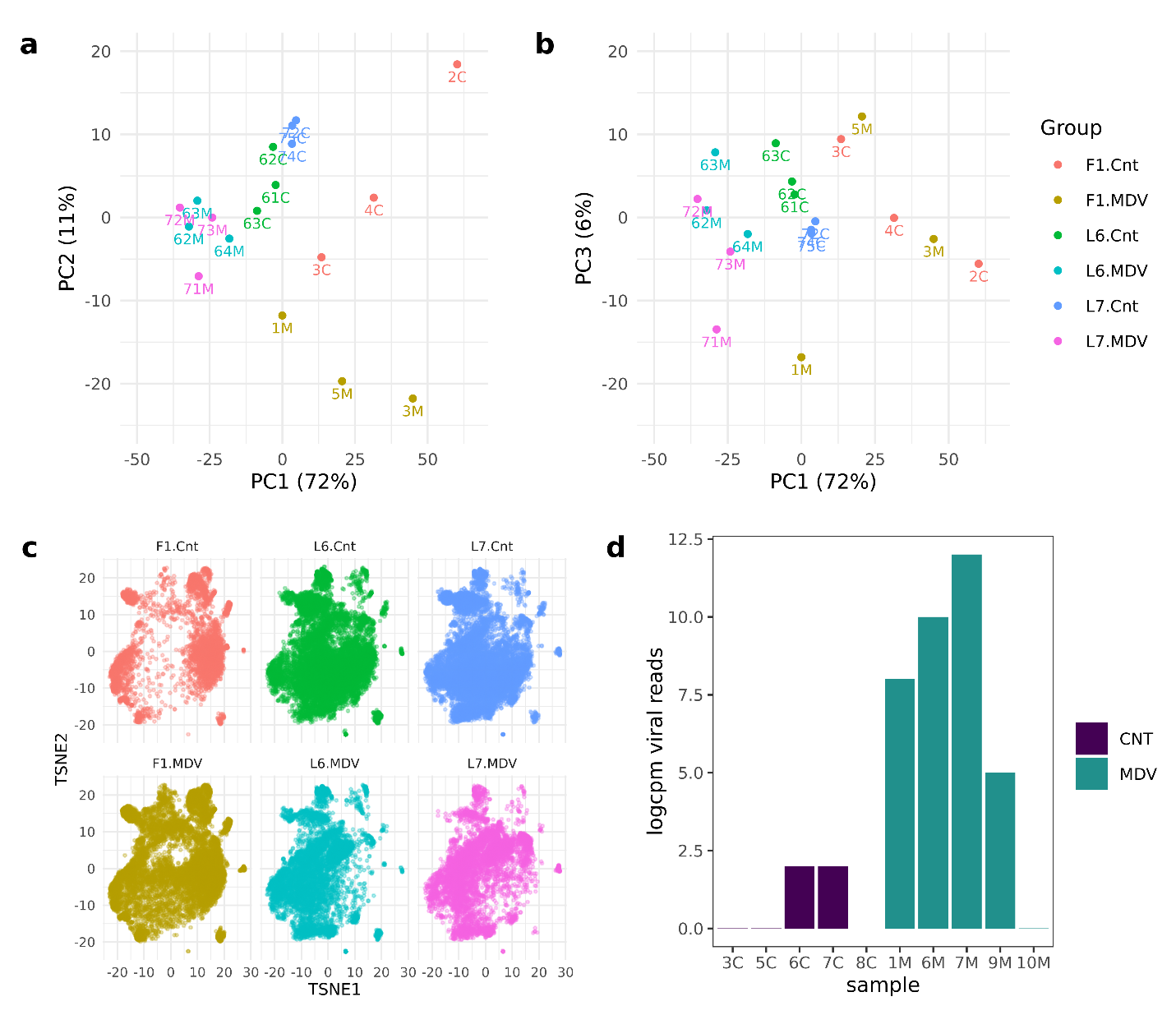


**Supp. Figure 1**. Principal components, TSNE clusters and Marek’s Disease viral reads. (a-b) Principal components analysis of the of the top 1000 variable genes for each line in infected and uninfected samples are shown with (a) PC1 vs PC2, and (b) PC1 vs PC3. (c) TSNE maps after batch correction with MNN provide a visual representation of the contributions from each line and treatment group to the GIM. (d) Log2 counts per million (logcpm) of MD viral reads quantified for 10 of the 20 samples included in the F1 challenge study. The viral reads were captured using whole genome bisulfite sequencing (WGBS). Due to limitations in sample quantities, only two of the birds (3C and 1M) with scRNAseq were also sent for WGBS.


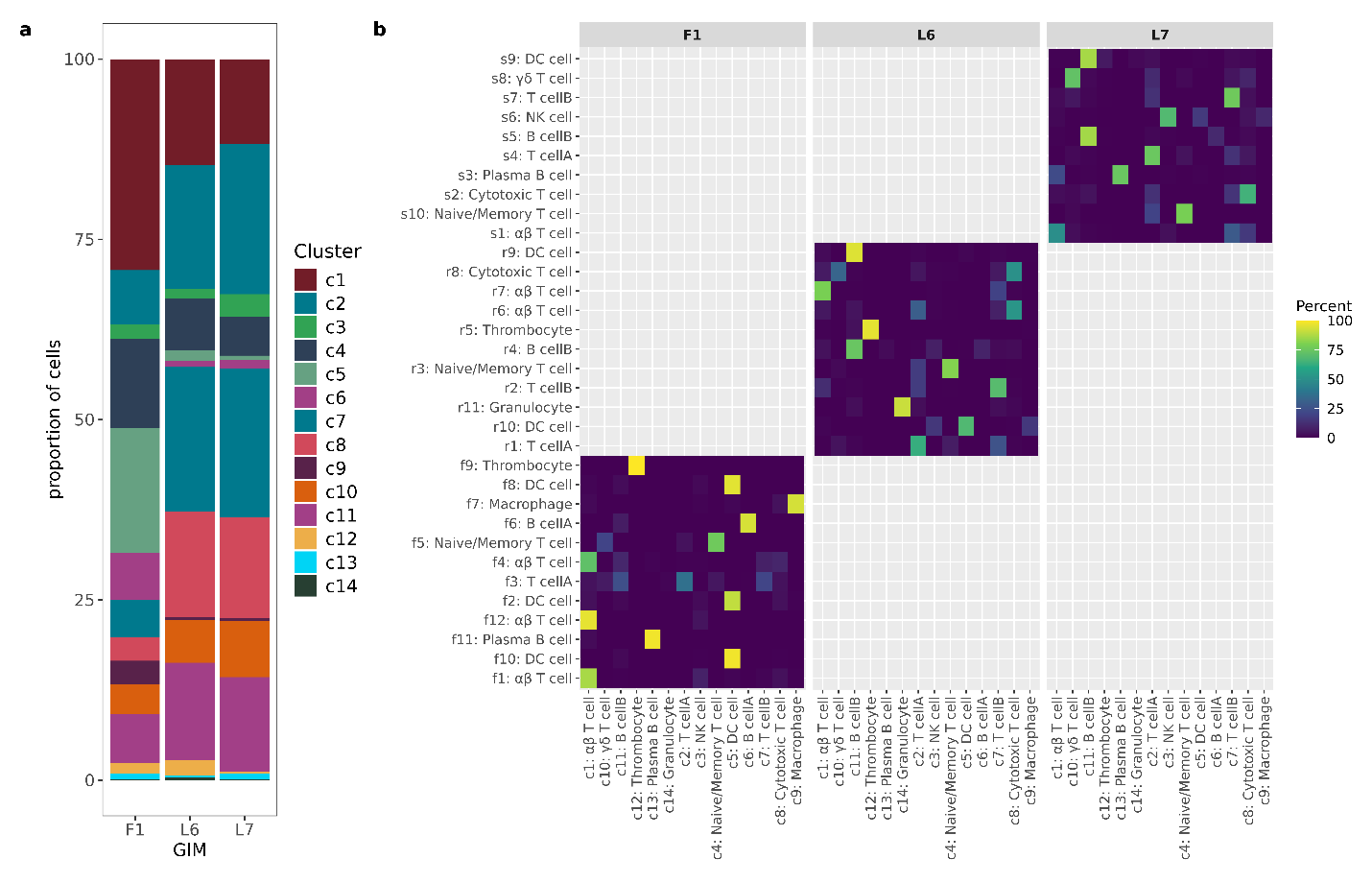


**Supp. Figure 2**. Proportion of cells in each cluster by each experimental line. (a) Percent of cells in each cluster by experimental line in the global immune map (GIM). Each cluster is represented with a different color based on the annotated cell type. The T cell (c2 and c7) and B cell (c6 and c11) clusters were the only cell types made of two clusters each. (b) Concordance plots depicting the percent of cells from the GIM clusters (x-axis) mapping to equivalent cell types in the Line specific Immune Maps (LIM, y-axis). Lighter colors represent a higher concordance between the GIM and LIM.


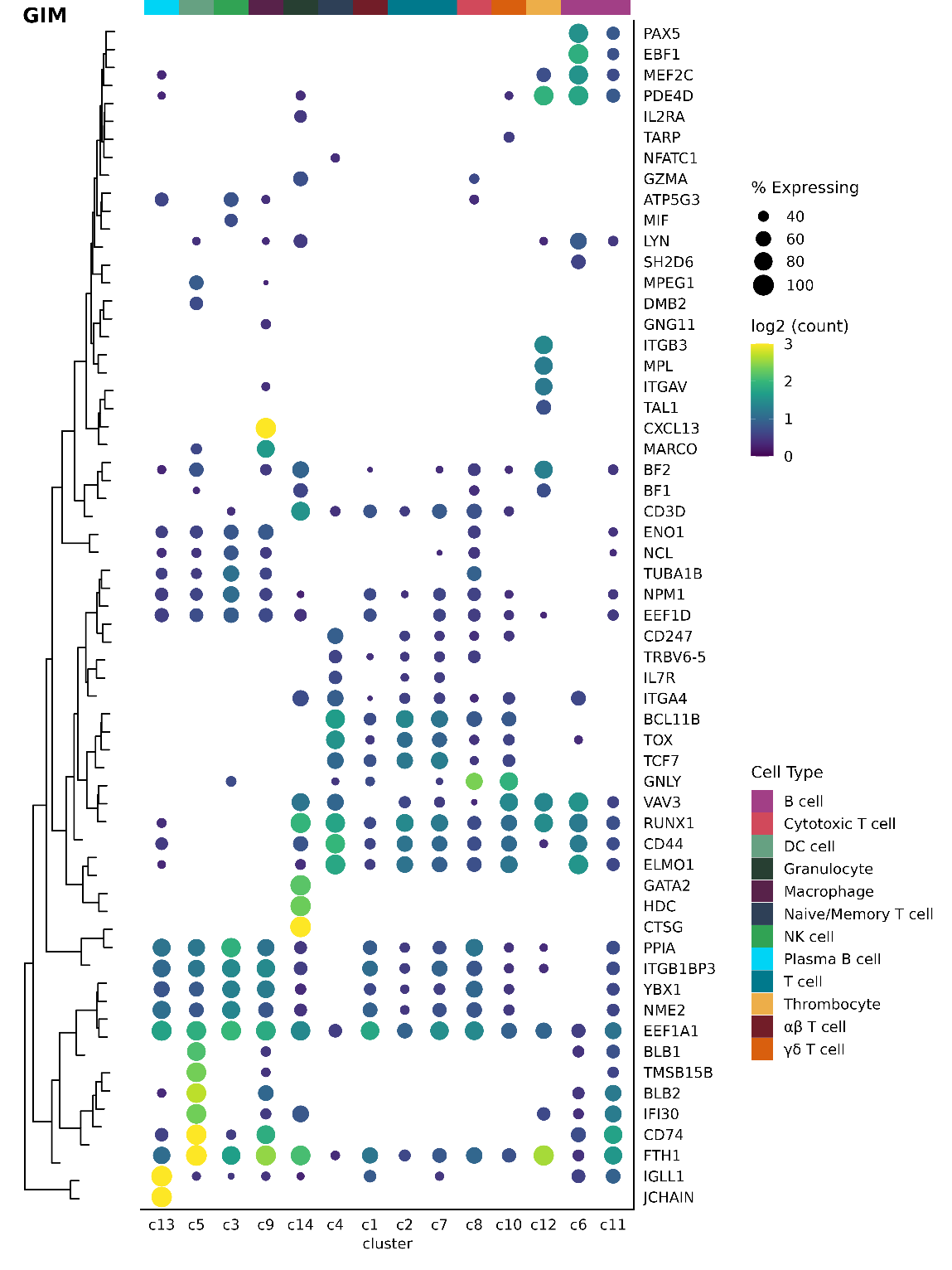


**Supp. Figure 3.** Global immune cell map gene signatures. Clusters were annotated to immune cell types based on the most upregulated genes within a cluster supplemented with expression levels of known immune cell type gene markers. The dot plot shows the percent of cells expressing the select gene, represented in the size of the dots, and the log2 average count identified with different hues from dark purple (lowly expressed) to yellow (highly expressed). The dendrograms on left hand side grouped genes together based on their expression facilitating the distinction of immune subtypes-based gene expression signatures.


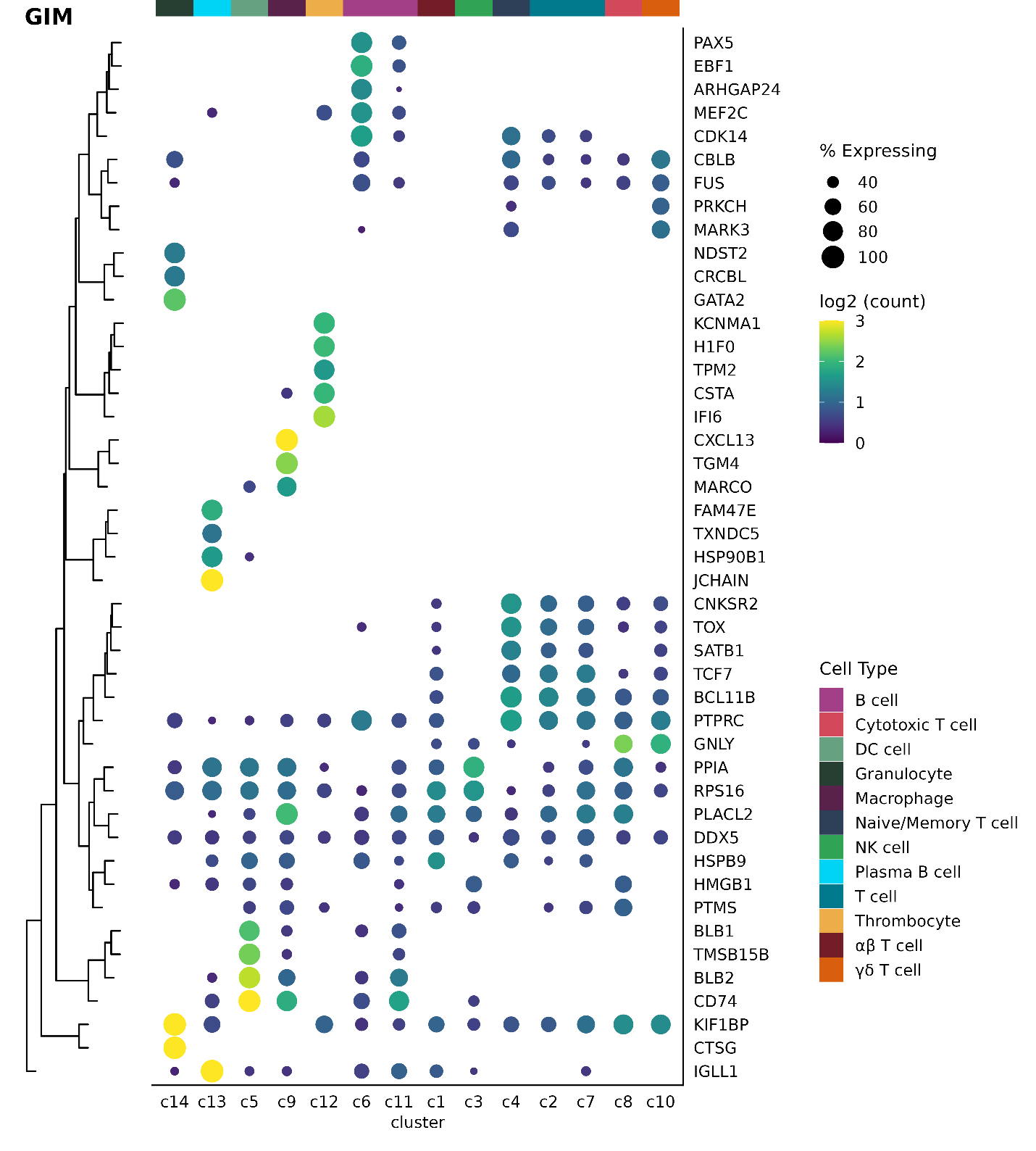


**Supp. Figure 4.** Top 5 highly expressed genes within each cluster in the GIM. The dot plot shows the percent of cells expressing the selected gene, represented in the size of the dots, and the log2 average count shown with different hues from dark purple (lowly expressed) to yellow (highly expressed) in the color of each dot.


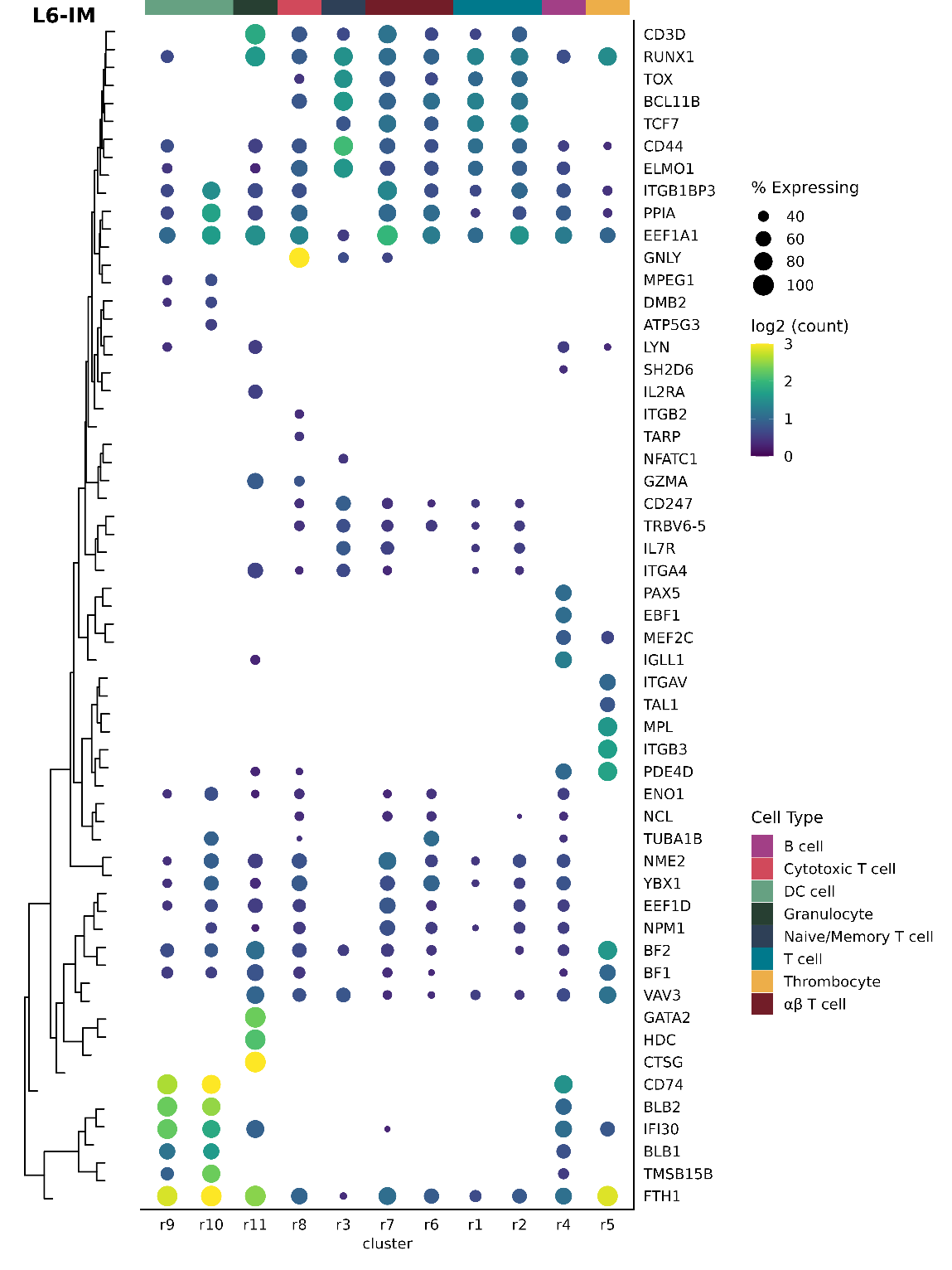


**Supp. Figure 5.** MD resistant Line 6 immune cell map gene signatures. Clusters were annotated to immune cell types based on the most upregulated genes within a cluster supplemented with expression levels of known immune cell type gene markers. The dot plot shows the percent of cells expressing the select gene, represented in the size of the dots, and the log2 average count identified with different hues from dark purple (lowly expressed) to yellow (highly expressed). The dendrograms on left hand side grouped genes together based on their expression facilitating the distinction of immune subtypes-based gene expression signatures.


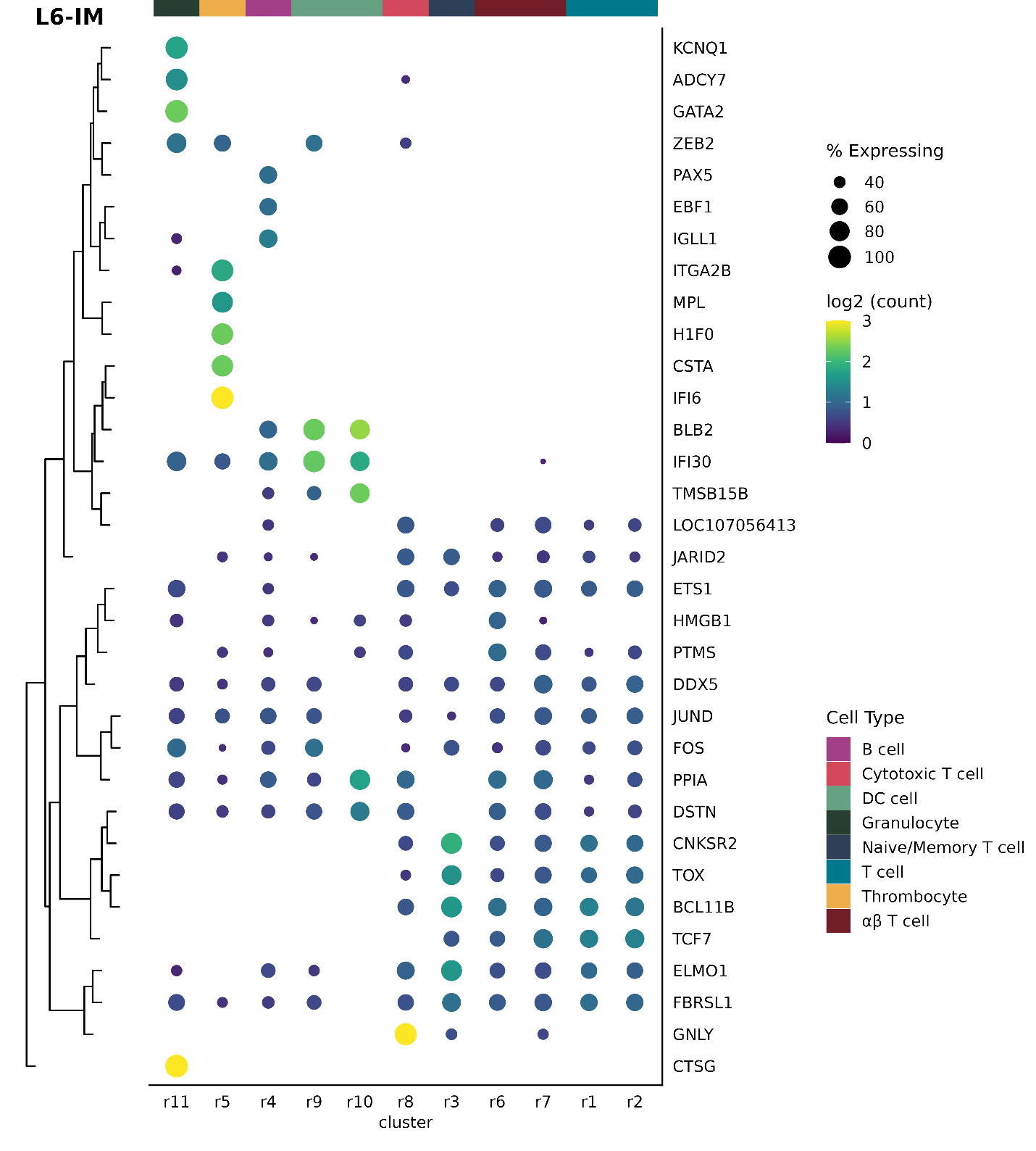


**Supp. Figure 6.** Top 5 highly expressed genes within each cluster in the L6-IM. The dot plot shows the percent of cells expressing the selected gene, represented in the size of the dots, and the log2 average count shown with different hues from dark purple (lowly expressed) to yellow (highly expressed) in the color of each dot.


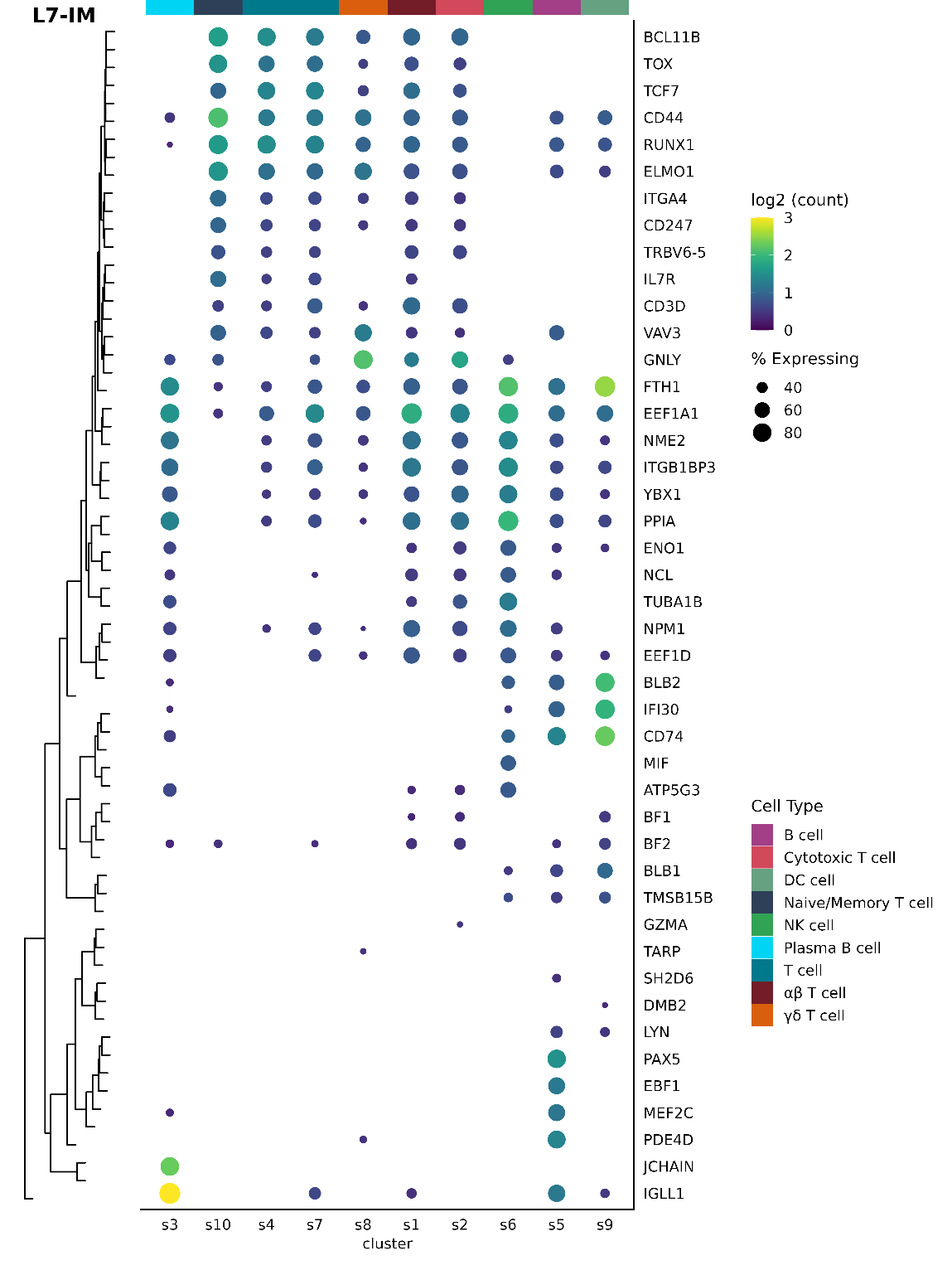


**Supp. Figure 7.** MD susceptible Line 7 immune cell map gene signatures. Clusters were annotated to immune cell types based on the most upregulated genes within a cluster supplemented with expression levels of known immune cell type gene markers. The dot plot shows the percent of cells expressing the select gene, represented in the size of the dots, and the log2 average count identified with different hues from dark purple (lowly expressed) to yellow (highly expressed). The dendrograms on left hand side grouped genes together based on their expression facilitating the distinction of immune subtypes-based gene expression signatures.


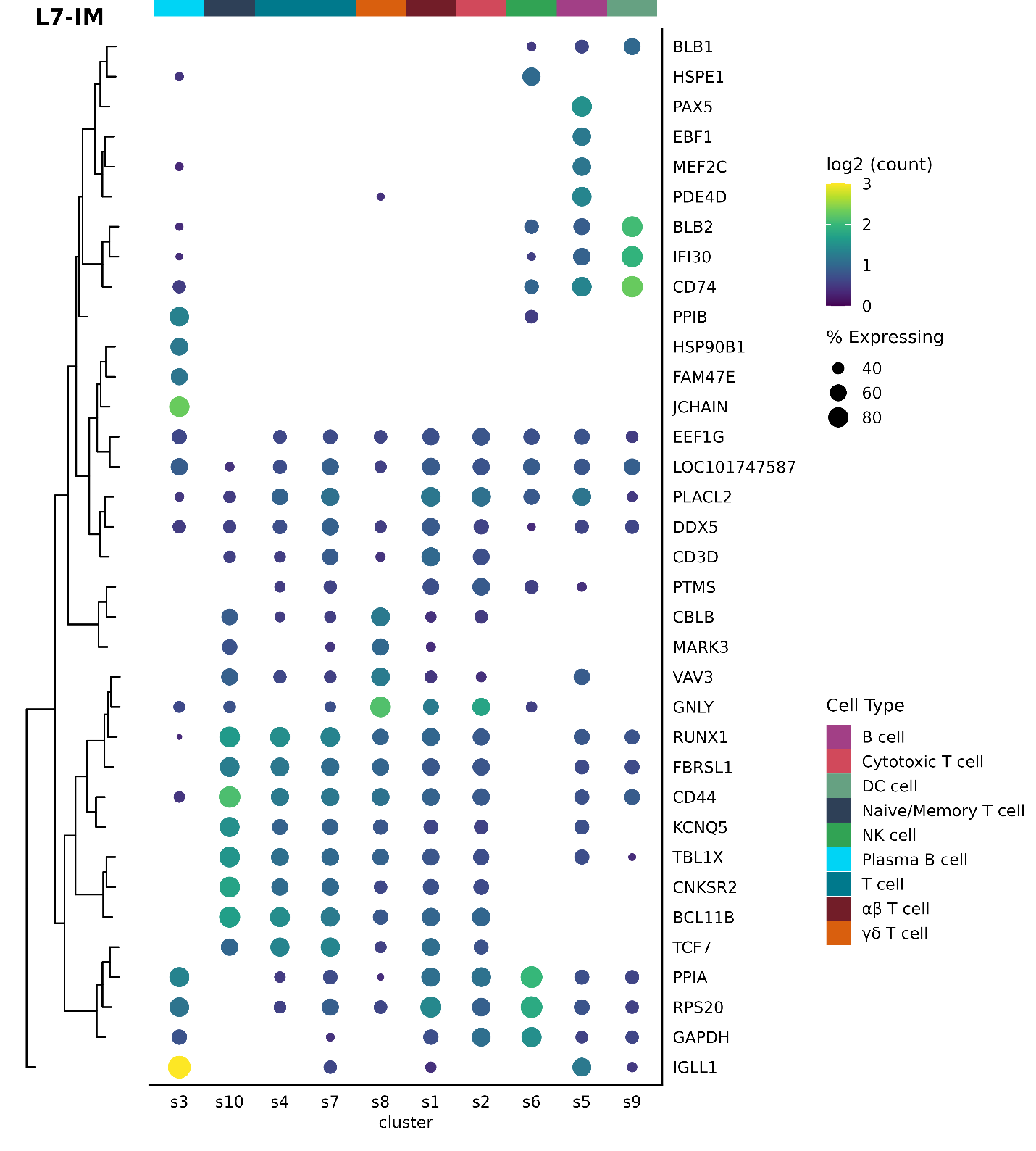


**Supp. Figure 8.** Top 5 highly expressed genes within each cluster in the L7-IM. The dot plot shows the percent of cells expressing the selected gene, represented in the size of the dots, and the log2 average count shown with different hues from dark purple (lowly expressed) to yellow (highly expressed) in the color of each dot.


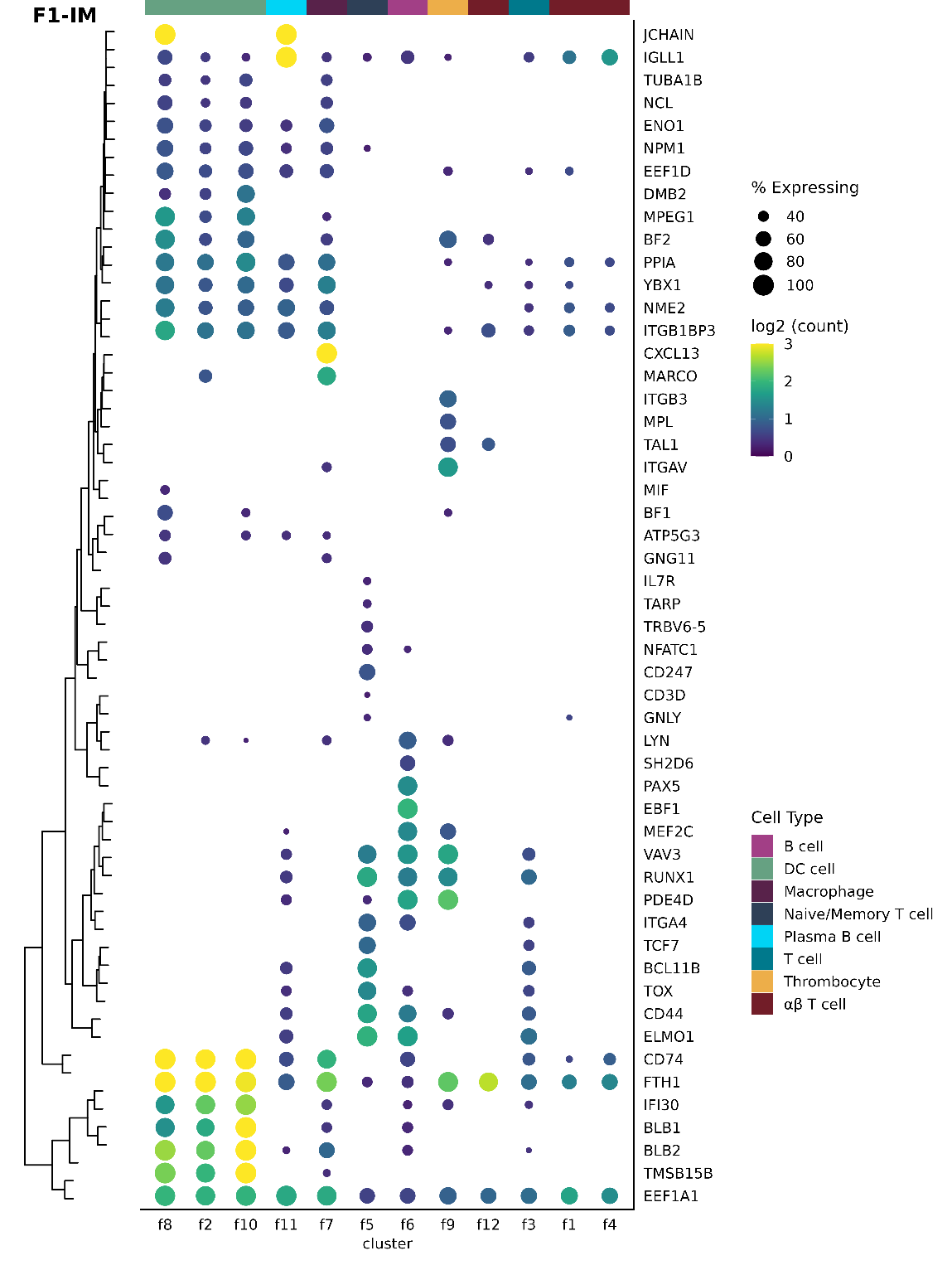


**Sub. Figure 9.** Immune cell map for F1 population gene signatures. Clusters were annotated to immune cell types based on the most upregulated genes within a cluster supplemented with expression levels of known immune cell type gene markers. The dot plot shows the percent of cells expressing the select gene, represented in the size of the dots, and the log2 average count identified with different hues from dark purple (lowly expressed) to yellow (highly expressed). The dendrograms on left hand side grouped genes together based on their expression facilitating the distinction of immune subtypes-based gene expression signatures.


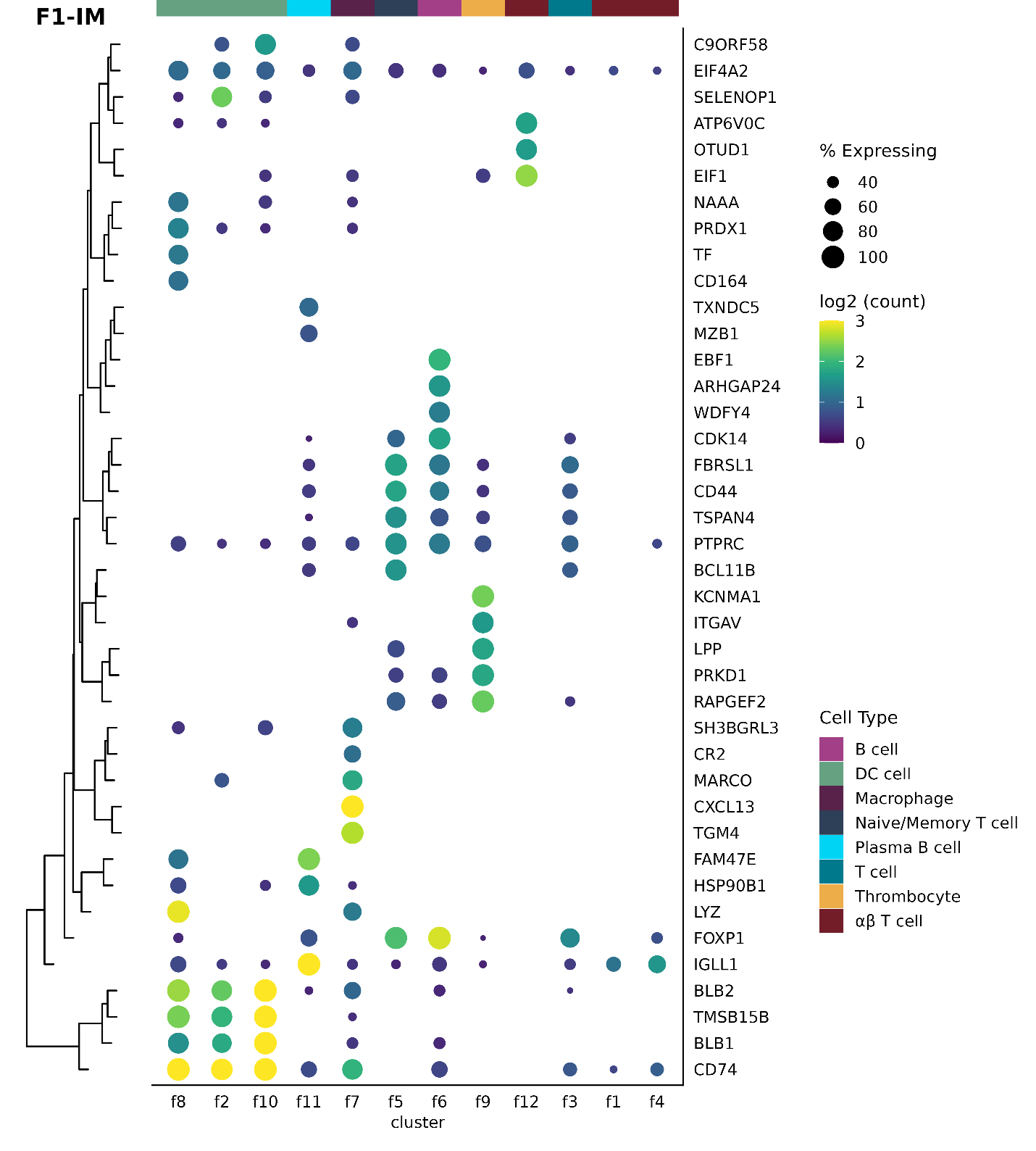


**Supp. Figure 10.** Top 5 highly expressed genes within each cluster in the F1-IM. The dot plot shows the percent of cells expressing the selected gene, represented in the size of the dots, and the log2 average count shown with different hues from dark purple (lowly expressed) to yellow (highly expressed) in the color of each dot.


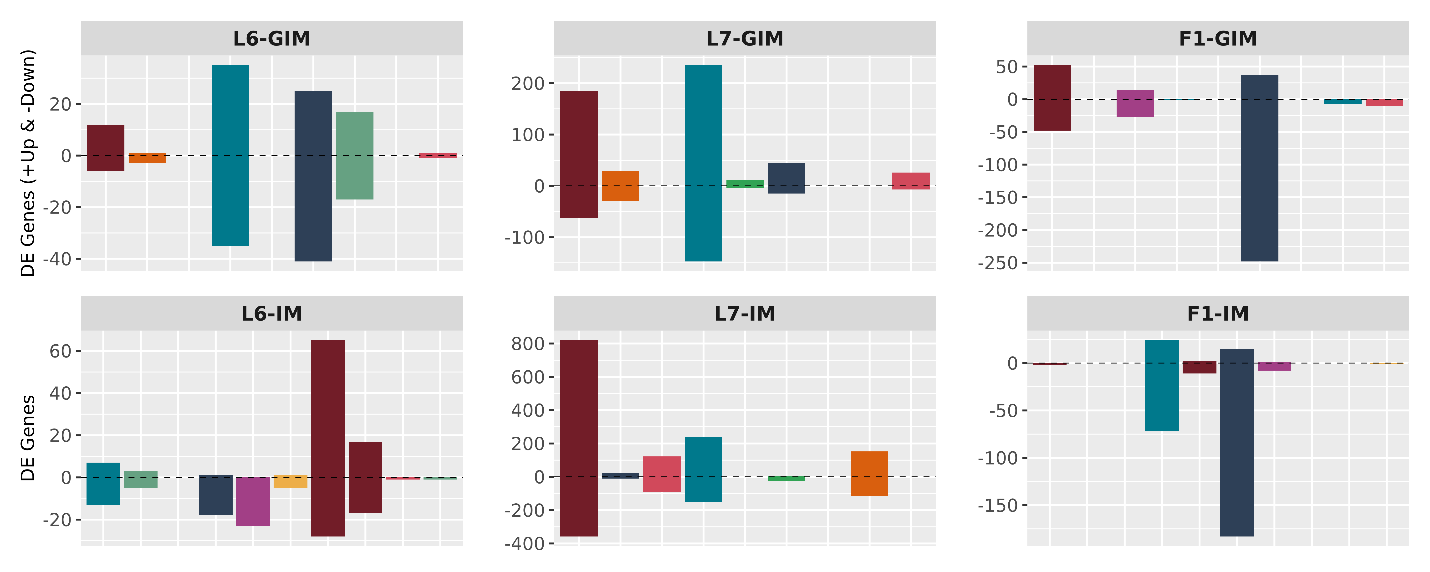


**Supp. Figure 11.** Number of differentially expressed genes per immune cell type. The top panel contains the results from the GIM and the bottom from the LIM.

**
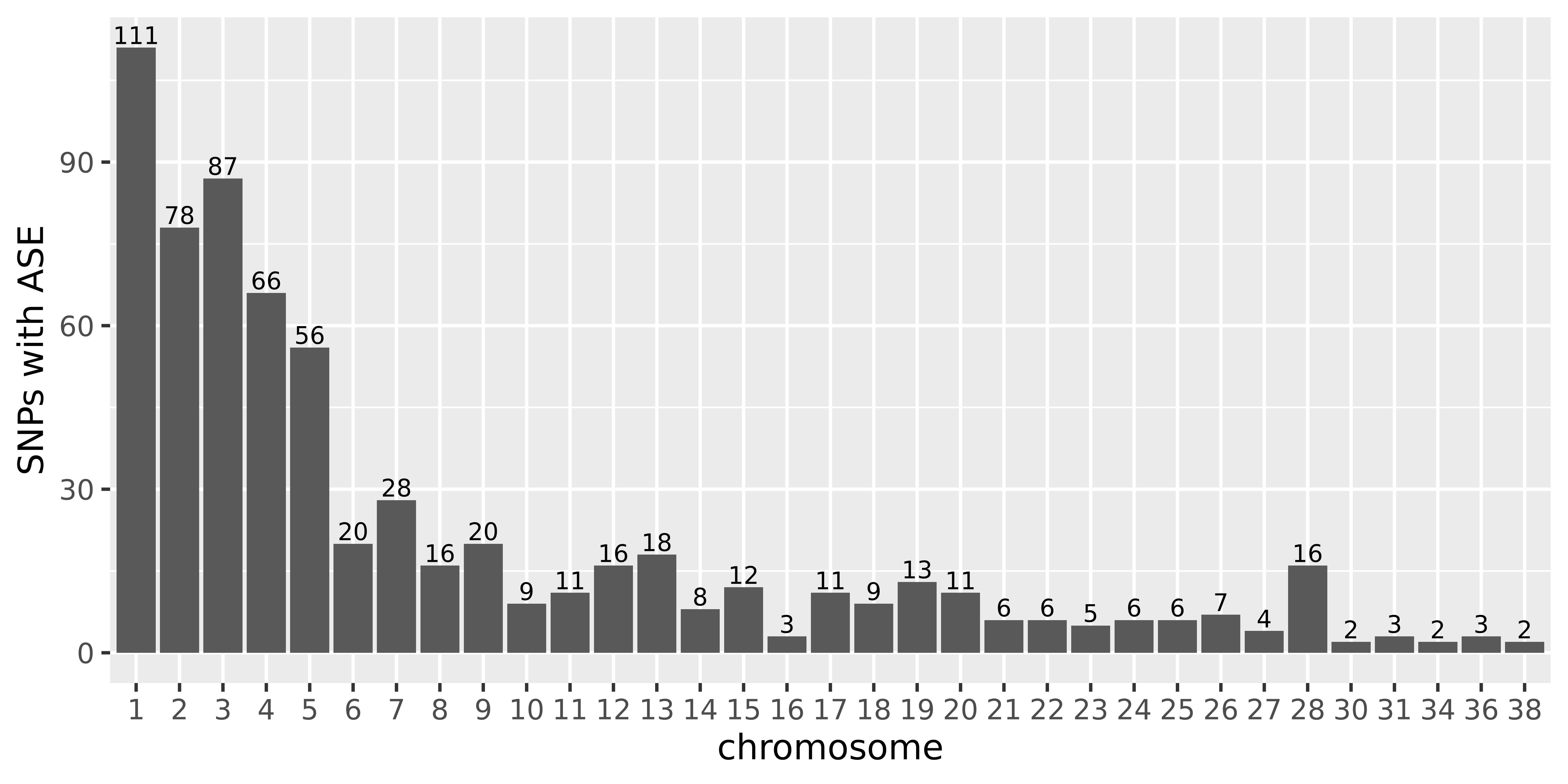
**

**Sub. Figure 12.** SNP exhibiting allele specific expression in F1 by chromosome. SNP called from quality filtered single cell transcriptomes


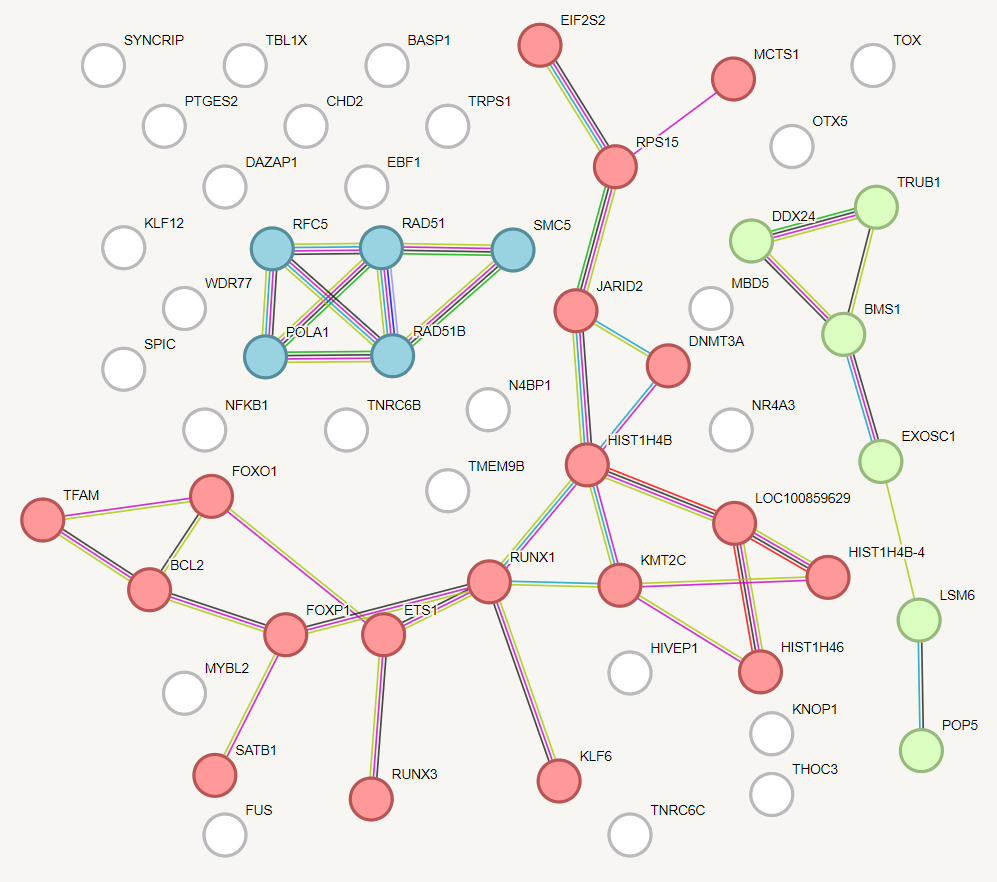


**Sub. Figure 13.** Candidate genes associated with MD resistance. Genes differentially expressed in specific immune cell types and mapped to a SNP exhibiting ASE were associated with nucleic acid binding activity. Kmeans clustering of a protein-protein interaction network associated with nucleic acid molecular functions. Cluster 1 contains genes that are a structural constituent of chromatin and the histone methyltransferase complex (19 genes, red). Cluster 2 contains genes that form part of the sno(s)RNA containing ribonucleoprotein complex (6 genes, green). Cluster 3 contains genes associated with homology directed repair through homologous recombination (3 genes, blue).
